# Using in-cell SHAPE-Seq and simulations to probe structure–function design principles of RNA transcriptional regulators

**DOI:** 10.1261/rna.054916.115

**Published:** 2016-06

**Authors:** Melissa K. Takahashi, Kyle E. Watters, Paul M. Gasper, Timothy R. Abbott, Paul D. Carlson, Alan A. Chen, Julius B. Lucks

**Affiliations:** 1 School of Chemical and Biomolecular Engineering, Cornell University, Ithaca, New York 14850, USA; 2 Department of Chemistry and RNA Institute, University at Albany, Albany, New York 12222, USA

**Keywords:** RNA transcription regulation, RNA structure/function, synthetic biology, SHAPE-Seq

## Abstract

Antisense RNA-mediated transcriptional regulators are powerful tools for controlling gene expression and creating synthetic gene networks. RNA transcriptional repressors derived from natural mechanisms called attenuators are particularly versatile, though their mechanistic complexity has made them difficult to engineer. Here we identify a new structure–function design principle for attenuators that enables the forward engineering of new RNA transcriptional repressors. Using in-cell SHAPE-Seq to characterize the structures of attenuator variants within *Escherichia coli*, we show that attenuator hairpins that facilitate interaction with antisense RNAs require interior loops for proper function. Molecular dynamics simulations of these attenuator variants suggest these interior loops impart structural flexibility. We further observe hairpin flexibility in the cellular structures of natural RNA mechanisms that use antisense RNA interactions to repress translation, confirming earlier results from in vitro studies. Finally, we design new transcriptional attenuators in silico using an interior loop as a structural requirement and show that they function as desired in vivo. This work establishes interior loops as an important structural element for designing synthetic RNA gene regulators. We anticipate that the coupling of experimental measurement of cellular RNA structure and function with computational modeling will enable rapid discovery of structure–function design principles for a diverse array of natural and synthetic RNA regulators.

## INTRODUCTION

RNAs are powerful regulators of gene expression. Natural RNA regulatory mechanisms are diverse and include RNAs that control transcription, translation, and mRNA degradation (Gottesman 2004; Winkler and Breaker 2005; Brantl 2007). Some of these natural RNA regulators act as switches in response to the presence of various factors such as antisense RNAs (Wagner and Simons 1994; Franch et al. 1999; Brantl 2007), small molecules (Winkler and Breaker 2005), and proteins (Sugiyama and Nakada 1967; Gottesman 2004). Many of these natural examples have been further engineered or used as inspiration for controlling gene expression in synthetic biology applications that range from optimizing metabolic pathways to developing biocontainment strategies (Sharma et al. 2011; Callura et al. 2012; Na et al. 2013; Farasat et al. 2014; Pardee et al. 2014; Gallagher et al. 2015). While directly engineering natural regulators for improved function within these applications is showing promise, our incomplete understanding of the complex mechanisms underlying many natural RNA regulators hinders our ability to quickly design them de novo. The puzzling nature of these mechanisms has sparked an increased interest in uncovering RNA structure–function design principles that can be used to efficiently engineer large libraries of RNA regulators with optimized (Green et al. 2014) and sometimes expanded function (Chappell et al. 2015a,b; Meyer et al. 2015).

**FIGURE 1.**
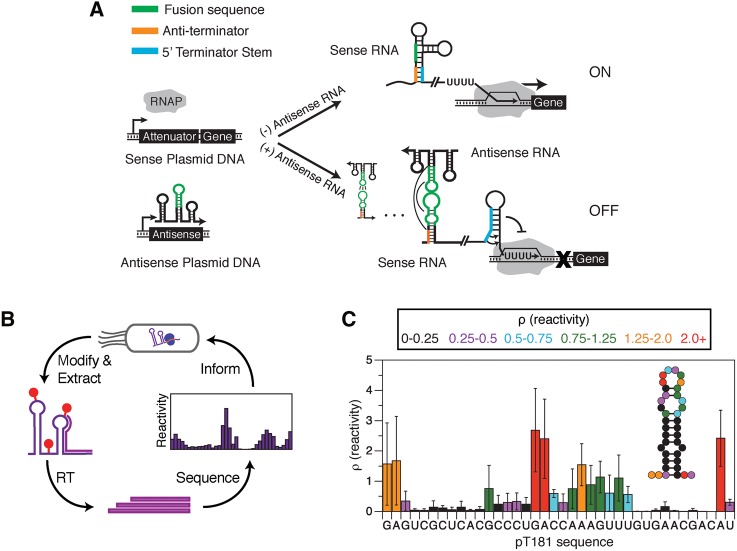
Using in-cell SHAPE-Seq to uncover structure–function design principles for chimeric RNA transcriptional attenuators. (*A*) Regulation of transcription by chimeric attenuators. Chimeric transcriptional attenuators are engineered by replacing portions of the *S. aureus* pT181 transcriptional attenuator (Novick et al. 1989; Brantl and Wagner 2000) with RNA-binding regions (fusion sequences) from natural antisense-RNA translational regulators (Takahashi and Lucks 2013). In the absence of antisense RNA, the anti-terminator sequence sequesters the 5′ portion of the terminator stem, preventing terminator formation and allowing transcription elongation by RNA polymerase (RNAP, ON). When antisense RNA is present, its interaction with the attenuator sequesters the anti-terminator, thus allowing terminator formation and preventing downstream transcription (OFF). Antisense/attenuator binding initiates as a kissing hairpin interaction that proceeds to a more extensively paired state, shown schematically with interaction lines (Brantl and Wagner 2000). (*B*) In-cell SHAPE-Seq (Watters et al. 2016) overview. In-cell SHAPE-Seq characterizes cellular RNA structures using a SHAPE chemical probe that preferentially modifies nucleotides in flexible regions of the RNA. After modification in culture, RNA is extracted, followed by reverse transcription (RT), next-generation sequencing, and bioinformatics steps that yield information about cellular RNA structures in the form of SHAPE reactivity profiles. (*C*) In-cell SHAPE-Seq reactivity profile for the sensing hairpin of the pT181 attenuator in *E. coli*. Reactivity profile and restrained secondary structure prediction is shown for the first hairpin of the pT181 attenuator. Color-coded reactivity spectrum represents an average of three independent in-cell SHAPE-Seq experiments, with error bars representing standard deviations at each nucleotide. High reactivities indicate unpaired or unrestrained nucleotides. The hairpin structure (*inset*) represents the minimum free energy structure generated by RNAstructure (Reuter and Mathews 2010) using average in-cell SHAPE-Seq reactivity data as restraints.

Of the many natural RNA regulatory mechanisms available, RNA-responsive transcriptional attenuators are particularly versatile (Lucks et al. 2011b; Takahashi and Lucks 2013). These types of attenuators are RNA transcriptional repressors that have important functions in nature and were initially discovered through their role in plasmid copy number control mechanisms (Novick et al. 1989). They function as transcriptional switches that prevent transcription elongation when an antisense small RNA (sRNA) is present (Fig. 1A; Novick et al. 1989; Brantl and Wagner 2000). Because they regulate downstream RNA synthesis as a function of an RNA input, attenuators can also be used in synthetic, RNA-only transcription networks that propagate signals directly as RNA molecules (Lucks et al. 2011b; Takahashi et al. 2015). Attenuators also have the potential to simplify the construction of genetic networks by removing the need to express intermediate protein species. In particular, variants of the *Staphylococcus aureus* plasmid pT181 attenuator (Novick et al. 1989) have been used to construct a variety of genetic networks: In tandem they act as genetic logic gates (Lucks et al. 2011b; Chappell et al. 2015a), and in series they can be used to construct RNA-only transcriptional cascades (Lucks et al. 2011b) and single input modules (Takahashi et al. 2015). In addition, there is also evidence that these RNA networks propagate signals on the fast timescales governed by RNA degradation rates (Takahashi et al. 2015), giving them potential kinetic advantage over their protein-mediated counterparts.

Recently, we sought to expand upon these promising results of using attenuators to construct higher-order RNA genetic networks by engineering additional orthogonal variants of the pT181 attenuator that can serve as independently acting components of more sophisticated networks (Takahashi and Lucks 2013). The pT181 attenuator is an RNA sequence in the 5′ untranslated region (UTR) of a transcript that can fold into two different structures (Fig. 1A; Novick et al. 1989; Brantl and Wagner 2000). In the OFF structure, an intrinsic terminator hairpin forms that prevents transcription elongation of a downstream gene. In the ON structure, intramolecular interactions with an anti-terminator sequence prevent terminator formation and allow transcription elongation. Interactions with an antisense RNA that is completely complementary to the 5′ portion of the attenuator bias folding into the OFF state, leading to antisense RNA-mediated transcription attenuation (Novick et al. 1989; Brantl and Wagner 2000). Specifically, the antisense RNA interacts with a 5′ hairpin structure of the attenuator that contains the anti-terminator (Fig. 1A). Antisense binding is thought to occur first through a loop–loop kissing hairpin interaction that then proceeds to a more extensively paired state between the antisense RNA and the 5′ attenuator hairpin, which sequesters the anti-terminator sequence and allows the intrinsic terminator hairpin to form (Fig. 1A; Novick et al. 1989; Brantl and Wagner 2000). The key to engineering orthogonal attenuators is to change the specificity of the antisense–attenuator kissing hairpin interaction while maintaining the ability of the attenuator to form the terminated (OFF) and anti-terminated (ON) states. Efforts to rationally mutate the pT181 attenuator proved difficult, with only two orthogonal variants generated from a large number of nonfunctional mutants that only differed by several nucleotides from the wild-type sequence (Lucks et al. 2011b). We thus instead developed a strategy to create chimeric attenuators by replacing the portion of the pT181 attenuator that is hypothesized to nucleate RNA–RNA interactions with sequences from naturally occurring antisense RNA translational regulators (Fig. 1A; Takahashi and Lucks 2013). While we were successful in creating additional chimeric antisense/attenuator pairs that were orthogonal to the wild-type system, the process was mostly trial-and-error and required systematically varying the length of the translational regulatory sequence added for each new chimera. In addition, gene expression characterization indicated that many chimeras were not functional; showing reduced ON state expression, reduced repression in the presence of cognate antisense RNA, or both. We did however observe that every chimeric attenuator that functioned properly was predicted to contain interior loop structures in the hairpin of the attenuator that interacted with the antisense RNA (Takahashi and Lucks 2013).

Previous work has demonstrated the functional importance of interior loop structures within antisense RNA mechanisms that repress translation (Asano et al. 1991; Siemering et al. 1993; Hjalt and Wagner 1995a,b; Kolb et al. 2001b). In these systems, antisense binding initiates through a kissing hairpin interaction with structures on the sense target mRNA, proceeding to a stable four-way junction complex that ultimately results in translation inhibition (Siemering et al. 1994; Kolb et al. 2001a,b). For the copy number control system of plasmid R1, it was shown that eliminating interior loop structures with mutations in the sense and antisense RNAs significantly reduced their pairing rates in vitro (∼44–185-fold lower) (Hjalt and Wagner 1995a) and impaired function in vivo (Hjalt and Wagner 1995a,b). Further investigation of this system showed that removal of the interior loops prevented the formation of the four-way junction by stopping the interaction at the kissing complex (Kolb et al. 2001b). The importance of interior loop structures was also shown for the antisense RNAs of pMU720 (Siemering et al. 1993) and ColIB-P9 (Asano et al. 1991), both of which are thought to inhibit translation through a similar four-way junction complex (Siemering et al. 1994; Kolb et al. 2001a). While these works were performed on translational regulatory systems, the same principles may hold true for antisense-mediated transcriptional regulation. In fact, any reduction in the binding rate of antisense RNA could have a larger impact on in vivo function of transcriptional attenuators since the regulatory decision must be made during the fast timescales of active transcription (Brantl and Wagner 2000).

Therefore, we sought to investigate the role of interior loop structures in a series of engineered transcriptional attenuators by characterizing their structure and function with in-cell SHAPE-Seq and molecular dynamics simulations. In-cell SHAPE-Seq is a newly developed technique that combines in-cell chemical probing with next-generation sequencing to characterize RNA structures inside cells (Fig. 1B; Watters et al. 2016). In this measurement, SHAPE reagents introduced in cell cultures modify cellular RNAs at positions that are unstructured (Tyrrell et al. 2013; McGinnis and Weeks 2014). After RNA extraction, modification positions are identified through reverse transcription, which is blocked by the modification. Sequencing of the resultant cDNAs allows the calculation of a reactivity spectrum for the RNAs studied (Fig. 1C). High reactivities indicate RNA regions that are unstructured or flexible, while low reactivities typically indicate regions that are structured or participate in intermolecular interactions.

In-cell SHAPE-Seq analysis of functional and nonfunctional members of the chimeric attenuator library suggested that interior loop structures in the attenuator hairpin lead to high reactivities in the upper stem that are required for function. To confirm this observation, we made mutations predicted to close these interior loops along with complementary mutations to the antisense RNA and demonstrated that the loss of the interior loop reactivity resulted in a loss of attenuator function. SHAPE-Seq reactivity data and molecular dynamics simulations comparing a functional attenuator hairpin and one of the nonfunctional loop closures indicated that interior loops confer structural flexibility to the upper hairpin stems. We also confirmed that the interior loops present in the natural RNA translational regulator hairpins used to create the chimeric attenuators confer structural flexibility in the cell, though not to the same extent as in the chimeric attenuators. Finally, we show that interior loops can be used as a design principle to create new attenuators in silico that function as desired in vivo, thus expanding our capabilities to engineer RNA gene regulators.

## RESULTS

### In-cell SHAPE-Seq reveals highly reactive nucleotides in hairpin stems of functional attenuators

Previous work developing chimeric RNA transcription attenuators suggested that interior loops were required in the attenuator 5′ hairpin responsible for sensing antisense RNA (sensing hairpin) (Takahashi and Lucks 2013). In particular, every functionally repressive chimeric attenuator was predicted to contain interior loop structures in the upper portion of the sensing hairpin above the hairpin base that contains the anti-terminator sequence (Fig. 1A). We first sought to confirm the presence of these interior loops by repeating the functional characterization in Takahashi and Lucks (2013) and using in-cell SHAPE-Seq to probe the secondary structures of a series of chimeric attenuators that were predicted to have a varying number of interior loops.

To characterize chimeric attenuator function, plasmids were constructed where each attenuator was placed downstream from a constitutive promoter and upstream of the superfolder GFP (SFGFP)-coding sequence (Pédelacq et al. 2006) on a medium copy plasmid. Complementary antisense RNAs were placed on a separate high copy plasmid downstream from the same constitutive promoter (Supplemental Fig. S1). Each chimeric attenuator plasmid was transformed into *E. coli* TG1 cells along with either its cognate antisense or a no-antisense control plasmid (Supplemental Table S1). Individual colonies were picked, grown overnight, sub-cultured into minimal media and grown until logarithmic growth was reached. Fluorescence (FL) (485 nm excitation, 520 nm emission) and optical density (OD) (600 nm) were measured for each culture (see Materials and Methods).

To characterize chimeric attenuator structure, we followed the recently developed in-cell SHAPE-Seq protocol (Watters et al. 2016). Plasmids were constructed with truncated attenuator sequences placed downstream from a constitutive promoter and upstream of the synthetic ECK120051404 terminator (Chen et al. 2013) on a medium copy plasmid (Supplemental Fig. S1). To probe secondary structure, we added 1-methyl-7-nitroisatoic anhydride (1M7), or the control solvent dimethyl sulfoxide (DMSO), to *E. coli* TG1 cell cultures that had been transformed with attenuator plasmids. After RNA extraction, reverse transcription (RT) was performed with primers that target the ECK120051404 terminator (Watters et al. 2016). The resulting cDNAs were prepared for sequencing following the in-cell SHAPE-Seq protocol, sequenced on an Illumina MiSeq and analyzed by the standard SHAPE-Seq computational pipeline (Fig. 1B; Aviran et al. 2011a,b; Lucks et al. 2011a; Loughrey et al. 2014). The output of an in-cell SHAPE-Seq experiment is a reactivity spectrum for a specific RNA that indicates the scaled probability that each nucleotide in the RNA was modified by 1M7 in the cell. Flexible nucleotides such as those in single-stranded regions are more likely to be chemically modified and therefore have higher reactivities (Fig. 1C).

**FIGURE 2.**
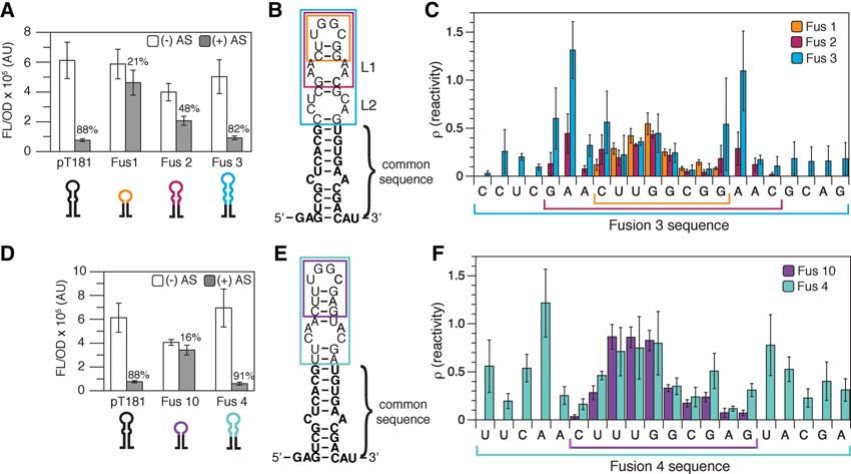
In-cell SHAPE-Seq reveals structural differences between functional and nonfunctional chimeric attenuators. (*A*) Functional characterization of chimeric fusions between pT181 and interaction sequences from the pMU720 (Siemering et al. 1994) regulator sequence. Average fluorescence (FL/OD) of *E. coli* TG1 cells with (+ AS) or without (− AS) antisense RNA. Error bars represent standard deviations of nine biological replicates. (*B*) In-cell SHAPE-restrained secondary structure prediction of the first attenuator hairpin comparing the sequences of Fusions 1–3. Bold nucleotides indicate the common sequence across all fusions originally from the pT181 attenuator. Colored boxes indicate the sequence from the pMU720 regulator included in Fusions 1–3 (cartoons under *A*). (*C*) In-cell SHAPE-Seq reactivity comparison for Fusions 1–3 (Fus 1–3). Plot showing a comparison of reactivities for the sequences derived from pMU720 included in Fusions 1–3, indicated by colors as in *B*. Reactivity spectra represent an average of three independent in-cell SHAPE-Seq experiments with error bars representing standard deviations at each nucleotide. Colored brackets denote the nucleotides included in each fusion. Full reactivity spectra can be found in Supplemental Figure S2. (*D*–*F*) As in *A*–*C* but for characterization of chimeric fusions between pT181 and interaction sequences from the R1 (Persson et al. 1988) regulator sequence. Full reactivity spectra can be found in Supplemental Figure S4.

Structural and functional characterization was performed on a set of chimeric attenuators that contained three different fusion sequence lengths from the pMU720 translational regulator (Fig. 2A–C; Siemering et al. 1994; Takahashi and Lucks 2013). As had been observed previously, functional characterization of these attenuators demonstrated that Fusions 1 and 2 function poorly, with measured repression of 21% and 48%, respectively. On the other hand, the 82% repression observed with Fusion 3 was comparable to the repression of the original pT181 system (Fig. 2A). This is in contrast to a thermodynamic folding analysis of the ON and OFF structural states for each attenuator, which predicts that all three fusions should be functional since the OFF state with antisense bound was predicted to be more stable than the ON state (Supplemental Table S7). We then compared the in-cell SHAPE-Seq reactivity spectra for the three fusions. As expected, the reactivities of the common sequence derived from the original pT181 attenuator and shared by all of the chimeras were very similar across the three fusions (Supplemental Fig. S2). Additionally, the reactivities of the apical loop region were very similar across all three fusions. However, the reactivities differed within the fusion sequence in the upper stem region of the hairpin (Fig. 2C). Specifically, we observed high reactivities for Fusion 3 in the region of the predicted upper interior loop (L1) when compared to Fusion 2. Interestingly, we observed similar reactivity spectra for the fusion sequence of all of the corresponding antisense RNAs for Fusions 1–3 (Supplemental Fig. S3), indicating that the high reactivities in the upper stem of the sensing hairpin of the attenuator are more important for function.

To further test this, we performed a similar characterization of the chimeric attenuators created from the R1 translational regulator (Fig. 2D–F; Persson et al. 1988; Takahashi and Lucks 2013). Again we observed poor repression for Fusion 10 (16%), which is not predicted to contain an interior loop, and 91% repression for Fusion 4, which is predicted to contain an interior loop (Fig. 2D,E). A comparison of the in-cell SHAPE-Seq reactivity spectra for Fusions 10 and 4 showed very similar reactivities for the pT181 common sequence (Supplemental Fig. S4) and high reactivities in the interior loop and neighboring nucleotides for Fusion 4 (Fig. 2F). Together these data suggested a direct link between high reactivities in the upper interior loop region of the sensing hairpin of the attenuator, and proper attenuator function.

### Closing interior loops results in loss of attenuator function and decrease in hairpin reactivities

**FIGURE 3.**
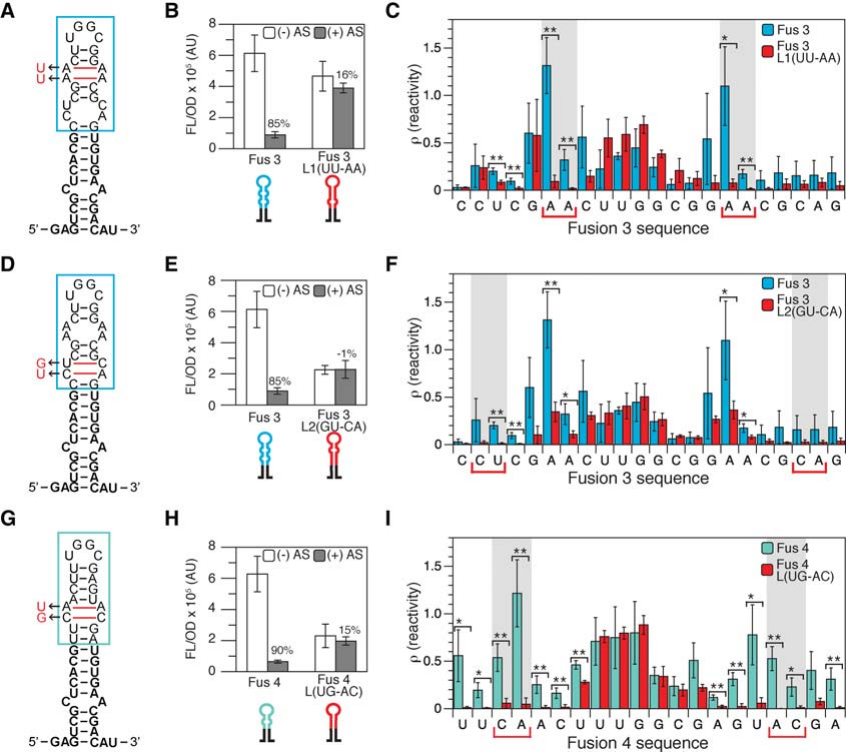
Mutations that close sensing hairpin interior loops break attenuator function and decrease hairpin reactivity. (*A*) In-cell SHAPE-restrained secondary structure prediction of the Fusion 3 hairpin indicating mutations to close the upper interior loop (L1, UU–AA). Boxed region indicates nucleotides shown in the reactivity spectra in *C*. (*B*) Functional characterization of Fusion 3 and the Fusion 3 L1 mutant that closes the top interior loop. Average fluorescence (FL/OD) of *E. coli* TG1 cells with (+ AS) or without (− AS) antisense RNA. Error bars represent standard deviations of nine biological replicates. (*C*) In-cell SHAPE-Seq reactivity comparison for Fusion 3 and Fusion 3 L1(UU–AA). Reactivity spectra represent an average of three independent in-cell SHAPE-Seq experiments with error bars representing standard deviations at each nucleotide. Shaded regions and colored brackets indicate nucleotides of the mutated interior loop. A Welch's *t*-test was performed comparing the reactivity at each nucleotide between Fusion 3 and Fusion 3 L1(UU–AA). (*) *P* < 0.1, (**) *P* < 0.05. (*D*–*F*) As in *A*–*C* but for the Fusion 3 L2 mutant that closes the bottom interior loop. (*G*–*I*) As in *A*–*C* but for the characterization of Fusion 4 and the Fusion 4 L mutant that closes the interior loop.

To further investigate the importance of interior loop structures for attenuator function, and to ensure that the increased reactivities were not simply a result of longer fusion sequences within the functional chimeric attenuators, we mutated bases in the chimeric sensing hairpin to close the interior loops of Fusions 3 and 4 along with complementary mutations to the antisense RNAs (Fig. 3A,D,G). We first closed the upper interior loop (L1) of Fusion 3 by mutating the 5′ side from AA to UU. The resulting Fusion 3 L1(UU–AA) was not functional, and showed only 16% repression when challenged with its completely complementary antisense RNA (mutated from the Fusion 3 antisense) (Fig. 3B). Corresponding to this loss of function was an almost complete loss of in-cell SHAPE-Seq reactivity for the nucleotides in the L1 interior loop (Fig. 3C). Similarly, closing the lower interior loop (L2) to create mutant Fusion 3 L2(GU-CA), resulted in this mutant showing no functional repression (−1%) with similar drops in reactivities for the affected nucleotides (Fig. 3E,F). Interestingly, the mutations in L2 also caused the reactivities in the L1 loop to drop to similar levels as those in the nonfunctional Fusion 2 (Supplemental Fig. S5), indicating that loop closures can have nonlocal effects on intramolecular interactions within the hairpin. It is also worth noting that Fusion 3 L2(GU–CA) showed a significant reduction in the ON expression level in the absence of antisense RNA. We observed similar results in loss of function and reduction in reactivities when these mutations were made to the 3′ half of the interior loops instead of the 5′ half (Supplemental Fig. S6).

We also tested interior loop closing mutations for Fusion 4 (Fig. 3G–I; Supplemental Fig. S7). Again, we observed a loss in attenuator function (15% repression) and near complete loss of reactivities for the affected nucleotides within the loop closure mutants Fusion 4 L(UG–AC) and Fusion 4 L(AC–UG) (Fig. 3H,I; Supplemental Fig. S7). Closing the interior loop in Fusion 4 also resulted in significantly lower reactivities for the neighboring nucleotides in the hairpin. In addition, Fusion 4 L(UG–AC) and L(AC–UG) also displayed significant reductions in ON expression level in the absence of their antisense RNAs, similar to Fusion 3 L2(GU–CA). Again, a comparison of the in-cell SHAPE-Seq reactivity spectra for Fusion 4 L(UG–AC) with the nonfunctional Fusion 10 showed that the interior loop closures have remarkably similar reactivity spectra to the fusions that do not include the interior loop sequence at all (Supplemental Fig. S5). These results confirm the requirement of high reactivities in the upper stem of the sensing hairpin of the attenuator for proper attenuator function. The nonlocal reductions in reactivity upon loop closure also suggest that high reactivities in the hairpin stem could be a result of overall hairpin flexibility, which could be necessary for proper antisense recognition and binding.

### Molecular dynamics simulations show loop closure mutations reduce hairpin fluctuations

To enhance the interpretation of our in-cell SHAPE-Seq reactivity data, we simulated structural fluctuations in the Fusion 3 and Fusion 3 L2(GU–CA) attenuator hairpins with replica exchange molecular dynamics (Supplemental Methods). This allowed us to investigate changes in hairpin structure and dynamics induced by closing the L2 interior loop. The resulting trajectories were analyzed for the presence or absence of base-pairing between each residue and its potential partner according to secondary structures predicted by RNAstructure (Reuter and Mathews 2010). Each time point of the simulation was assessed using the 3DNA base-pair search algorithm (Lu and Olson 2003), which uses strictly geometric criteria and counts any hydrogen bond between nucleotides as a base pair. This broad definition allows the identification of both canonical and noncanonical pairings and even captures transient interactions between neighboring residues. With these measures, we calculated the percentage of frames in the simulations in which each base pair of the hairpins was formed to investigate hairpin structure fluctuations (Supplemental Fig. S8A,B). Convergence was assessed by plotting the cumulative average base-pair occupancy of residue pairs in the fusion sequence (Supplemental Fig. S9) and statistical significance was assessed by calculating a bootstrap error estimate (Carlstein 1986).

**FIGURE 4.**
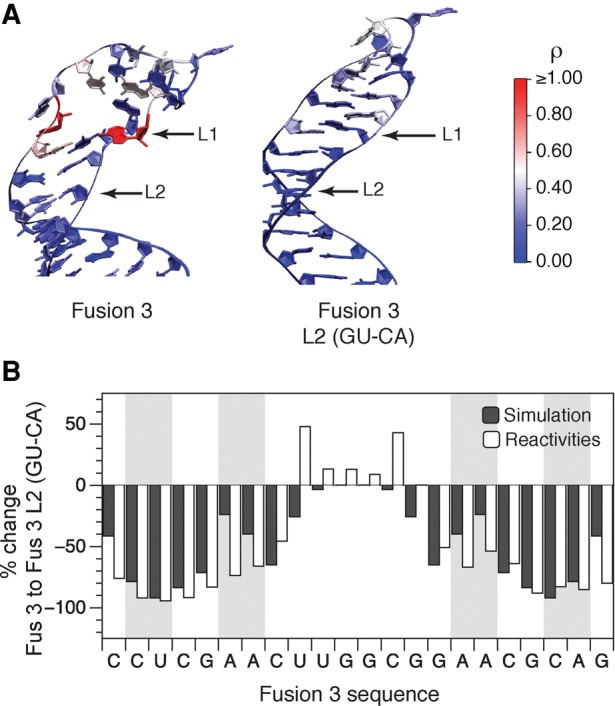
Comparison of in-cell SHAPE-Seq reactivities with molecular dynamics simulations suggests that hairpin interior loops confer structural flexibility. (*A*) Representative simulation structures of Fusion 3 and Fusion 3 L2(GU–CA). Nucleotides are overlaid with in-cell SHAPE-Seq reactivities from Figure 3F. (*B*) A comparison of percent change of simulated base-pair opening frequencies and in-cell SHAPE-Seq reactivities between Fusion 3 and Fusion 3 L2(GU–CA) reveals an increase in hairpin structural rigidity (drop in reactivities/opening frequencies) caused by interior loop closing mutations. The 3DNA base-pair search algorithm (Lu and Olson 2003) was used to calculate the percentage of frames in which each base pair in the hairpin was formed over 100 nsec for replica exchange molecular dynamics simulations of Fusion 3 and Fusion 3 L2(GU–CA) (Supplemental Fig. S8). This was then converted into percentages of frames in which each base pair was open (not occupied). This was then used to calculate a percent change in this value from Fusion 3 to Fusion 3 L2(GU–CA) and compared to a percent change in observed in-cell SHAPE-Seq reactivities for each nucleotide. Shaded regions represent nucleotides in the interior loops.

Simulations performed at 311 K were analyzed first as they approximately match the temperature of in-cell SHAPE-Seq experiments. The sampled hairpin conformational ensembles show higher base-pairing in the Fusion 3 L2(GU–CA) system, compared to Fusion 3 (Supplemental Fig. S8A). In addition, this increase in intra-strand contacts occurs not only as expected at the L2 mutation site, but also extends well into the hairpin including the nucleotides in the L1 interior loop as was observed through in-cell SHAPE-Seq reactivity changes for this mutant (Fig. 3F). The shift in the conformational ensemble of the Fusion 3 L2(GU–CA) hairpin toward more structured states is readily apparent upon visualizing individual simulation trajectories (Fig. 4A; Supplemental Fig. S10). This is even more apparent in simulations performed at 400 K (Supplemental Figs. S8, S10) and across the replica exchange temperature scale (Supplemental Movies 1, 2) where the Fusion 3 L2(GU–CA) fusion region even resists melting.

Since both the simulation results and in-cell SHAPE-Seq data suggested that closing the Fusion 3 L2 interior loop resulted in more constrained nucleotides in the L1 region, we sought to directly compare the simulation base-pair occupancies to SHAPE-Seq reactivities. Since high SHAPE-Seq reactivities are indicative of bases that are unpaired, we first converted the simulation results into simulated base-pair opening frequencies (Supplemental Fig. S8C,D). From this, we calculated a percent change in this measure from the Fusion 3 results to the Fusion 3 L2(GU–CA) results and compared to a percent change in in-cell SHAPE-Seq reactivities for the two constructs (Fig. 4B). It should be noted that the simulations only calculate pairing between nucleotides, and since pairs do not form in the apical loop of either hairpin, a percent base-pair occupancy change was not detected in the apical loops. However, since in-cell SHAPE-Seq reactivities measure the accessibility of those nucleotides to the SHAPE reagent, a percent change in reactivities between the two fusions was observed even though the raw reactivity values are similar (Fig. 3F). Outside of the apical loop, the comparison of percent change from Fusion 3 to Fusion 3 L2(GU–CA) between the simulation and experimentally measured reactivity changes showed remarkable agreement in the upper stem, suggesting that high reactivities in hairpin stems can be interpreted in terms of hairpin conformational flexibility. Thus the nonlocal drops in reactivities observed when closing the L2 interior loop can be interpreted as promoting base-pair formation in the loop adjacent positions, resulting in an extended helical structure (Fig. 4A; Supplemental Fig. S10). These base pairs would eventually have to be broken to form a loop–loop junction with the antisense RNA, effectively increasing the energetic barrier for the interaction, therefore suggesting a mechanism for the loss of function for this mutant.

### Natural kissing hairpin translational regulators contain similar flexible interior loops

**FIGURE 5.**
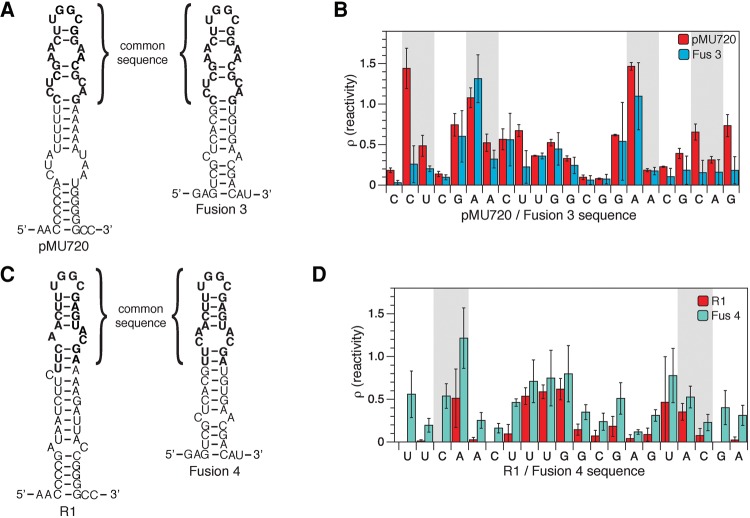
Hairpins from natural kissing hairpin translational regulators have similar nucleotide reactivity patterns as functional chimeric attenuator hairpins. (*A*) In-cell SHAPE-restrained secondary structure prediction of hairpins from the pMU720 regulator and the Fusion 3 chimeric attenuator. Bold nucleotides indicate the sequence from pMU720 used to create the Fusion 3 chimeric attenuator. (*B*) In-cell SHAPE-Seq reactivity spectra comparing pMU720 and Fusion 3 for their common nucleotides. Reactivity spectra represent an average of three independent in-cell SHAPE-Seq experiments with error bars representing standard deviations at each nucleotide. (*C*,*D*) As in *A*,*B* but for hairpins from the R1 regulator and the Fusion 4 chimeric attenuator. Bold nucleotides indicate the sequence from R1 used to create the Fusion 4 chimeric attenuator. Full reactivity spectra can be found in Supplemental Figure S11.

Next, we sought to examine whether or not having flexible interior loops is a general design principle for RNA regulatory mechanisms that utilize kissing hairpin interactions with antisense RNAs. Specifically, we examined the natural antisense RNA-mediated translational regulators that we used as sources to create chimeric attenuators. These natural translational regulators use interactions with antisense RNAs to trigger structural changes that block a ribosome binding site (Persson et al. 1988; Siemering et al. 1994). We used in-cell SHAPE-Seq to characterize the structures of the sensing hairpins for the pMU720 and R1 translational regulators that were used to create Fusions 3 and 4, respectively (Fig. 5; Supplemental Fig. S11). The in-cell SHAPE-Seq reactivity spectra for the sequence shared between the pMU720 and Fusion 3 sensing hairpins are statistically similar for all but one of the nucleotides in the apical loop and the first interior loop (Fig. 5B). The pMU720 hairpin shows additional flexibility in the lower interior loop likely due to the difference in sequence context for the bottom portion of the hairpin. The in-cell SHAPE-Seq reactivity spectra for the R1 hairpin also showed a flexible upper stem, though not as flexible as Fusion 4 (Fig. 5D). We note that the reactivity-restrained predicted secondary structure of the R1 hairpin suggested the formation of a nonsymmetrical interior loop with an additional single nucleotide bulge (Fig. 5C), which may explain these reduced reactivities when compared to Fusion 4. Together these results suggest that flexible hairpin structures due to interior loops could be a general design principle for kissing hairpin antisense-RNA interactions utilized by both natural and synthetic RNA regulatory mechanisms.

### In silico design of chimeric attenuators using interior loop design principles

Since all of our results suggested that interior loops and hairpin flexibility were required for attenuator function, we next sought to computationally design a chimeric attenuator using an interior loop as a structural requirement. To do this, we used the Nucleic Acid Package (NUPACK) webservers (Zadeh et al. 2011a). NUPACK uses thermodynamic analysis to predict secondary structures and interactions of dilute solutions of nucleic acid strands. Additionally, NUPACK is capable of designing nucleic acid sequences for one or more interacting strands given desired secondary structures and complexes (Zadeh et al. 2011b).

**FIGURE 6.**
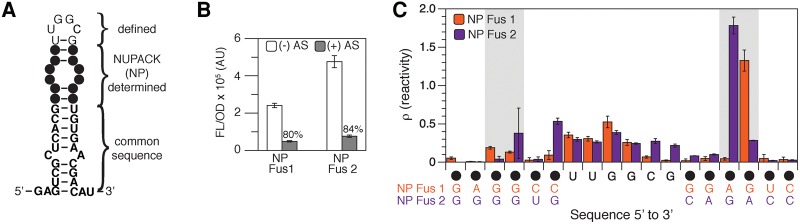
Using NUPACK to design chimeric attenuators with defined interior loops. (*A*) NUPACK (Zadeh et al. 2011b) design constraints. The nucleotides specified in the base of the hairpin are the same as those in the fusions from this study. The nucleotides specified in the apical loop are the same as those in Fusion 3 and 4. Filled circles represent nucleotides that NUPACK was allowed to design (Supplemental Note). (*B*) Functional characterization of two NUPACK (NP) designed fusions. Average fluorescence (FL/OD) of *E. coli* TG1 cells with (+ AS) or without (− AS) antisense RNA. Error bars represent standard deviations of nine biological replicates. (*C*) In-cell SHAPE-Seq reactivity spectra for the upper portion of NP Fusion 1 and 2 hairpin stems. Reactivity spectra represent an average of three independent in-cell SHAPE-Seq experiments with error bars representing standard deviations at each nucleotide. Shaded regions indicate nucleotides of the designed interior loop. Full reactivity spectra can be found in Supplemental Figure S13.

We chose the Fusion 4 hairpin secondary structure as a starting point for designing chimeric attenuators with NUPACK. Variations of the predicted secondary structure of the Fusion 4 sensing hairpin (Fig. 2E) were used as the target structure for the design algorithm. We specified the identities of the nucleotides at the base of the stem to be those common to all of our chimeric attenuators. This included the sequence of the anti-terminator at the base of the hairpin, which is necessary for proper switching. Additionally, the nucleotides in the apical loop were defined to be those of the apical loop in Fusion 3 and 4, leaving the interior loop and remaining upper stem nucleotides to be defined by NUPACK (Fig. 6A). Two designs for the structure in Figure 6A were generated, built, and tested with our functional assay. Both designs resulted in functional attenuators that display 80% and 84% repression (Fig. 6B). In-cell SHAPE-Seq analysis of the two NUPACK designed attenuators reveals one highly reactive nucleotide in the 3′ half of the interior loop in each. This is in contrast to Fusions 3 and 4 where the reactivities in the interior loops were symmetric (Fig. 2C,F). It is interesting that both NUPACK designed interior loops differ only by the position of a G residue on the 3′ side of the interior loop, which is the highly reactive nucleotide in each case. This suggests that in both cases the 3′ A residue is involved in an A–G base pair with the second G on the 5′ side stabilizing the pair, resulting in flexibility of the 3′ G. The flexibility of this single base was sufficient for proper attenuation in the cell. These results demonstrate the successful in silico design of functional transcriptional attenuators using an interior loop structural requirement.

In a different design approach, we specified the identities of the nucleotides at the base of the stem to be those common to all of our chimeric attenuators and allowed the bases between the interior and apical loops to vary (Supplemental Fig. S12A). Two designs were generated, built, tested with our functional assay, and analyzed with in-cell SHAPE-Seq. Both designs resulted in relatively poor attenuators with 19% and 44% repression (Supplemental Fig. S12B). However, in-cell SHAPE-Seq analysis showed relatively low reactivities for the interior loops and hairpin stem of the attenuator that showed the least repression (Supplemental Fig. S12C). The reactivity spectra for the attenuator that showed 44% repression did show higher reactivities, indicating more flexibility, which could be the reason for the improved repression seen for this attenuator. From these results, we concluded that hairpin flexibility is required for proper attenuator repression, but that not all interior loops result in structural flexibility.

## DISCUSSION

In this study, we used in-cell SHAPE-Seq experiments and molecular dynamics simulations to show that interior loops confer structural flexibility within the antisense RNA-sensing hairpins of chimeric transcriptional attenuators. It was also shown that this hairpin flexibility is required for attenuator function. By systematically comparing in-cell SHAPE-Seq reactivity spectra between functional and nonfunctional chimeric attenuators from a previous study (Takahashi and Lucks 2013), as well as with reactivity spectra for designed attenuator mutants, we determined that interior loops present in functional attenuators result in high in-cell SHAPE-Seq reactivities in the upper stem of the sensing hairpins (Figs. 2, 3). Molecular dynamics simulations of a functional attenuator and a nonfunctional mutant demonstrated that interior loops confer structural flexibility to the hairpins, allowing them to sample conformations that open the upper hairpin stem. In contrast, the mutant with the interior loop closure was found to be structurally rigid and not able to sample open conformations during the simulations. A comparison of the change in the simulated hairpin opening frequencies to the change in measured in-cell SHAPE-Seq reactivities between the functional and nonfunctional attenuators revealed a striking agreement between these two analyses (Fig. 4). Our results, together with previous work investigating antisense RNA translational regulators (Siemering et al. 1993; Hjalt and Wagner 1995a,b; Kolb et al. 2001b), suggest that interior loops may be a general design principle for kissing hairpin antisense RNA interactions. Indeed we were able to engineer new transcriptional attenuators in silico using this structural requirement (Fig. 6).

We also provide direct evidence that interior loops are present in the hairpins of antisense RNA regulators inside the cell as indicated by their high SHAPE-Seq reactivities (Figs. 2, 5). In addition, molecular dynamics simulations suggest that the interior loops act nonlocally to confer greater structural flexibility throughout the upper portion of the hairpin stem (Fig. 4). This was corroborated by our in-cell SHAPE-Seq results that showed nonlocal reductions in reactivities when the interior loops were closed (Fig. 3F,I). Previous work on the antisense RNA CopA from the R1 plasmid provided evidence of its interior loops conferring hairpin flexibility in vitro (Hjalt and Wagner 1995b). Interestingly, our probing results of the sense RNA (CopT) of the R1 system showed that the overall reactivities of the upper stem were less than what we observed in our chimeric transcriptional attenuator that used the same sequence (Fig. 5D). This suggests that in the cell our transcriptional attenuators are even more flexible than CopT, which may facilitate even more rapid binding and overall transcriptional repression in the cellular environment.

The data presented also provide evidence for the mechanism and importance of interior loops within antisense-mediated transcriptional regulatory systems, which are being shown to be increasingly important for synthetic biology applications (Lucks et al. 2011b; Chappell et al. 2015a; Takahashi et al. 2015). Initial in vitro structural analysis of the pT181 attenuator suggested that the 5′ hairpin of the pT181 sense RNA forms one large apical loop, therefore the importance of interior loops was not investigated (Brantl and Wagner 2000). Recent in vitro (Lucks et al. 2011a) and in-cell (Fig. 1C) SHAPE-Seq analysis of the attenuator suggests otherwise, and that an interior loop may be forming after all. Additionally, in vitro binding analysis of the pT181 regulator was inconclusive as to whether a full RNA duplex between sense and antisense was required for regulation (Brantl and Wagner 2000). Given our ability to engineer chimeric versions of the pT181 transcriptional attenuator using sequences from the R1 and pMU720 translational regulators, our chimeric regulators could proceed through a four-way junction intermediate (Kolb et al. 2000, 2001b). Perhaps a stable binding intermediate between the flexible parts of the antisense and attenuator loops is sufficient for regulation. Further analysis would be needed to confirm this hypothesis.

Interestingly, we observed that reactivities in the apical loops, where antisense kissing hairpin interactions are thought to initiate, were lower than those of the interior loops of the hairpins. This could be the result of the loop nucleotides being present in stacked conformations that better facilitate initial antisense recognition, which is in contrast to the high reactivities observed in loops that participate in interactions with single-stranded RNAs (Watters et al. 2016). In addition, we did not observe dramatic changes in the loop reactivities when the interior loops were closed suggesting that the overall loop structure is independent of the interior loops. More work is needed to understand the functional significance of this structural observation.

Hairpin flexibility may also be important for other aspects of transcriptional attenuation. The mechanism of pT181-based attenuators is complex, and not only requires efficient binding of antisense RNA to form the OFF state, but also requires the ability to refold into the anti-terminated ON state (Fig. 1A). In several cases, we actually observed that a loss in hairpin flexibility resulted in a decreased ON expression state. This indicates that flexibility in the sensing hairpin, which contains the anti-terminator sequence, is also required for proper refolding into the ON state. This suggests a dual role for hairpin flexibility to both sense and respond to incoming antisense RNAs, but also allow necessary structural transitions that form the basis of this molecular switch.

The previous work on dissecting the role of interior loops on translational repressor function had suggested that interior loops should be incorporated into artificial antisense RNA design (Hjalt and Wagner 1995a). Indeed, interior loops were a key structural feature used in the successful engineering of synthetic RNA translational activators (Green et al. 2014). By showing that interior loops play similar structural and functional roles in transcriptional attenuators, our work also suggests that they are important features for the design of new attenuators. We demonstrated that this is true by using NUPACK to design new hairpins with interior loop structures that resulted in new regulators with nearly identical repression in the presence of antisense RNA as the wild-type system (Fig. 6B). Interestingly, a comparison of NP Fusion 1 and 2 to Fusion 4 show a different pattern in SHAPE-Seq reactivities where the NUPACK attenuators each have one highly reactive nucleotide in the 3′ half of the interior loop versus a more symmetric reactivity profile for Fusion 4 (Supplemental Fig. S13). Additionally, the highly reactive nucleotide in NP Fusion 1 and 2 also differs in position. These results indicate that hairpin flexibility leading to a functional attenuator can be achieved in different ways.

Throughout this work, we also found remarkable agreement between changes in in-cell SHAPE-Seq reactivities and changes in calculated base-pairings from molecular dynamics simulations. In fact, the close agreement in these changes between Fusion 3 and the loop closure mutant L2 (GU–CA) gave strong evidence that reactivities can be interpreted in terms of time averaged nucleotide fluctuations. Indeed previous work has used SHAPE reactivities as restraints to guide molecular dynamics simulations to model the three-dimensional structures of RNAs (Gherghe et al. 2009; Ding et al. 2012). We anticipate this insight to play an important role in interpreting SHAPE and other chemical probing reactivities in future structural analysis on the broad array of RNAs on which SHAPE is increasingly applied (Lee et al. 2014).

Finally, we note that our strategy of comparing RNA regulator functional data to structural information provided by in-cell SHAPE-Seq to uncover cellular structure–function relationships is completely general and can be applied to other RNA systems (Watters et al. 2016). We anticipate this approach to enable rapid discovery of RNA structure–function design principles for a diverse array of regulators in nature and to accelerate the engineering of RNAs for a broad array of synthetic biology applications (Chappell et al. 2015b).

## MATERIALS AND METHODS

### Plasmid construction

A table of all the plasmids used in this study can be found in Supplemental Table S1, with key sequences provided in Supplemental Tables S2–S4. A schematic of the plasmid constructs used in this study is found in Supplemental Figure S1. The pT181 sense, antisense, and no-antisense control plasmids were constructs pAPA1272, pAPA1256, and pAPA1260, respectively, from Lucks et al. (2011b). The Fusion 1–4 and 10 plasmids were from Takahashi and Lucks (2013). Inverse PCR was used to create the interior loop mutants and NUPACK fusions. Gibson Assembly (Gibson et al. 2009) was used to create the SHAPE-Seq constructs from the standardized platform in Watters et al. (2016).

### Strains, growth media, and in vivo bulk fluorescence measurements

All experiments were performed in *E. coli* TG1 cells [F′ traD36 lacIq Δ(lacZ) M15 pro A^+^B^+^/supE Δ(hsdM-mcrB)5 (r_k_^−^ m_k_^−^ McrB-) thi Δ(lac-proAB)]. Plasmid combinations were transformed into chemically competent TG1 cells, plated on LB + Agar plates containing 100 µg/mL carbenicillin and 34 µg/mL chloramphenicol, and incubated overnight ∼17 h at 37°C. The plates were taken out of the incubator in the morning and left at room temperature for ∼7 h at which point three colonies were picked to inoculate 300 µL of LB containing carbenicillin and chloramphenicol at the concentrations above in a 2-mL 96-well block (Costar 3960). Cultures were grown overnight, ∼17 h at 37°C at 1000 rpm in a Labnet Vortemp 56 benchtop shaker. Four microliters of this overnight culture were then added to 196 µL (1:50 dilution) of supplemented M9 minimal media (1×M9 minimal salts, 1 mM thiamine hydrochloride, 0.4% glycerol, 0.2% casamino acids, 2 mM MgSO_4_, 0.1 mM CaCl_2_) containing the antibiotics above and grown for 3 h at the same conditions as the overnight culture. One hundred microliters of this culture were then transferred to a 96-well plate (Costar 3631) containing 100 µL of phosphate buffered saline (PBS). Fluorescence (FL) (485 nm excitation, 520 nm emission) and optical density (OD 600 nm) were measured using a Biotek Synergy H1M plate reader.

### Bulk fluorescence data analysis

On each 96-well block there were two sets of controls—a media blank (M9) and *E. coli* TG1 cells that do not express SFGFP (transformed with JBL001 and JBL002—see Supplemental Table S1). The block contained three replicates of each control. Three independent transformations were performed on separate days with three colonies characterized per transformation (nine total). Average OD and FL were calculated for the nine replicates of each control. OD and FL for each colony were first corrected by subtracting the average value of the media blank. The ratio of FL to OD (FL/OD) was then calculated for each well (colony) and the average FL/OD of the TG1 cells without SFGFP was subtracted from each colony's FL/OD value. The nine corrected FL/OD values were averaged and error bars represent standard deviations. For each attenuator/antisense pair, attenuation (% repression) was calculated as the percent decrease in FL/OD of cells containing both the attenuator and antisense plasmids versus the FL/OD of cells containing the attenuator and no-antisense control plasmids.

### Molecular dynamics

All-atom, replica exchange molecular dynamics simulations were performed for the Fusion 3 and Fusion 3 L2(GU–CA) hairpins using the GROMACS software package version 5.0.4 (Pronk et al. 2013). The Amber-99 force field (Wang et al. 2000) ported to GROMACS by Sorin and Pande (2005) was used with modifications for nucleic acids introduced by Chen and García (2013). Initial, all-atom RNA structures were generated by the MC-Sym package (Parisien and Major 2008) using secondary structures generated by RNAstructure (Reuter and Mathews 2010) as input. Energy minimization and short temperature and pressure equilibration were performed for each system. Production simulations utilized temperature replica exchange to enhance conformational sampling (66 replicas spanning 290–435K), and were performed for 130 nsec per replica (a cumulative total of 8580-nsec simulation time for each system). The first 30 nsec of each replica were considered further equilibration time and the final 100 nsec were used for analysis. Details of the simulation are provided in the Supplemental Information.

Base-pair occupancy was determined using the 3DNA software (Lu and Olson 2003) which applies strictly geometric criteria to identify all possible base pairs, including both standard Watson–Crick and wobble base pairs as well as noncanonical pairings. The do_x3DNA (github.com/rjdkmr/do_x3dna) module was used to facilitate the processing of GROMACS trajectories by 3DNA. The sampling statistical inefficiency was calculated using the timeseries.py python module by Chodera et al. (2007) and used to identify uncorrelated time points from which the sampling error was estimated following the bootstrap method (Carlstein 1986) with 20 bootstrap samples.

### In-cell SHAPE-Seq

For a full description of the in-cell SHAPE-Seq protocol see Watters et al. (2016). A brief description of the Materials and Methods is below.

### Strains, growth media, and RNA expression for in-cell SHAPE-Seq

All in-cell SHAPE-Seq experiments were performed in *E. coli* TG1 cells. Plasmids were transformed into chemically competent TG1 cells plated on LB + Agar plates containing 100 µg/mL carbenicillin or 34 µg/mL chloramphenicol and incubated overnight, ∼17 h at 37°C. The plates were taken out of the incubator in the morning and left at room temperature for ∼7 h at which point, colonies were picked to inoculate 500 µL of LB + antibiotic at the concentrations above in a 2 mL 96-well block (Costar 3960). Cultures were grown overnight, ∼17 h at 37°C at 1000 rpm in a Labnet Vortemp 56 benchtop shaker. Twenty-four microliters of this overnight culture were then used to subculture into 1.2 mL of LB + antibiotic. The subculture was grown under the same conditions for three hours before performing structure probing.

### RNA modification

Two aliquots of 500 µL from each subculture were made into separate wells on the 96-well block. The aliquots were modified with either 13.3 µL of 250 mM 1-methyl-7-nitroisatoic anhydride (1M7) in DMSO (6.5 mM final) (+) or 13.3 µL DMSO (−) for 3 min on the benchtop shaker.

### RNA extraction

Both modified (+) and control (−) samples were pelleted, then resuspended in 100 µL of hot Max Bacterial Enhancement Reagent (Life Technologies) before extraction with TRIzol Reagent (Life Technologies) according to the manufacturer's protocol. Extracted RNA was dissolved in 10 μL of water.

### Reverse transcription

For each extracted RNA sample, 3 µL of 0.5 µM oligonucleotide primer were added for reverse transcription (RT) (Supplemental Table S5). All RNAs were denatured at 95°C for 2 min, then 65°C for 5 min. After denaturing, each RNA sample was then snap-cooled on ice for 1 min before extension with Superscript III (Life Technologies) at 52°C for 25 min. After RT the RNA was hydrolyzed with 1 µL 10 M NaOH. The solution was then partially neutralized with 5 µL of 1 M hydrochloric acid, and ethanol precipitated.

### Adapter ligation

The cDNA from each RT reaction was separately ligated to a ssDNA adapter for Illumina sequencing with CircLigase I ssDNA ligase (Epicentre). Each ligation reaction was incubated at 60°C for 2 h, followed by deactivation at 80°C for 10 min. The ligated cDNA was then ethanol precipitated and dissolved in 20 µL of water. Unligated oligonucleotides were removed by purification with 36 µL of Agencourt AMPure XP beads (Beckman Coulter) according to the manufacturer's protocol.

### Quality control

Each single-stranded cDNA library was PCR amplified with Phusion polymerase (NEB) for 15 cycles with two forward primers, a selection primer (containing a sequence specific to the ECK120051404 terminator and part of the forward Illumina adapter) and a longer primer containing all of the forward Illumina adapter, and a fluorescent reverse primer that binds to the reverse Illumina adapter sequence as part of the ligated ssDNA adapter (Supplemental Table S5). The fluorescently tagged amplifications were run on an ABI 3730xl Analyzer with GeneScan 500 LIZ standard (Life Technologies) and checked for the correct full-length product (indicating good RT and PCR) and minimal side product formation.

### dsDNA library construction

Libraries passing quality analysis were PCR amplified with Phusion polymerase (NEB) for 15 cycles using three primers: a forward primer that contained an Illumina adapter, another forward selection primer specific to the ECK120051404 terminator, and a reverse primer that contained the other Illumina adapter and one of 18 TruSeq indexes (Supplemental Table S5). Excess primer was removed with ExoI (NEB) before purification with 90 µL of Agencourt AMPure XP beads (Beckman Coulter) according to the manufacturer's protocol.

### Next-generation sequencing

The molarity of the individual libraries was estimated from the lengths and intensity of peaks in the fluorescent quality traces, and the concentration of each library measured with a Qubit fluorometer (Life Technologies). All libraries were mixed to have the same final molar concentration and sequenced with an Illumina MiSeq v3 kit using 2 × 35-bp paired end reads.

### Data analysis

Reactivity spectra were calculated using Spats v0.8.0 and a number of utility scripts to prepare the Illumina output for Spats following previous work (Loughrey et al. 2014). Illumina adapter sequences were trimmed from each read using the FASTX toolkit (http://hannonlab.cshl.edu/fastx_toolkit/) and then aligned to the target RNA sequences with Bowtie 0.12.8 (Langmead et al. 2009) based on the input sense and antisense RNAs to determine locations of modifications. Spats separates the (+) and (−) channel reads according to the handle sequence, and calculates θ for each nucleotide using statistical corrections for RT drop-off, where θ represents the probability of modification at a particular nucleotide (Aviran et al. 2011b). Resulting θ values were then normalized to ρ values according to Watters et al. (2016).

### Structure folding predictions

RNA secondary structure predictions were performed using RNAstructure (Reuter and Mathews 2010). In-cell SHAPE-Seq reactivities (ρ) were used to restrain predictions with the pseudo-free-energy parameters m (1.1) and b (−0.3) (Loughrey et al. 2014) where indicated.

## SUPPLEMENTAL MATERIAL

Supplemental material is available for this article.

## Supplementary Material

Supplemental Material
