# Supplementary material for: Using in-cell SHAPE-Seq and simulations to probe structure–function design principles of RNA transcriptional regulators
Source: RNA. 2016 Jun;22(6):920–33. doi: 10.1261/rna.054916.115 (PMC4878617; doi:10.1261/rna.054916.115)
Supplement: Supplemental Material [file supp_22_6_920__index.html]

Using in-cell SHAPE-Seq and simulations to probe structure–function design principles of RNA transcriptional regulators — Using in-cell SHAPE-Seq and simulations to probe structure–function design principles of RNA transcriptional regulators — Supplemental Material 

# Using in-cell SHAPE-Seq and simulations to probe structure–function design principles of RNA transcriptional regulators

## Supplemental Material

- Supplemental\_Movie1.mp4 - mp4 file
- Supplemental\_Material.pdf - pdf file
- Supplemental\_Movie2.mp4 - mp4 file
