# Supplementary material for: Using in-cell SHAPE-Seq and simulations to probe structure–function design principles of RNA transcriptional regulators
Source: RNA. 2016 Jun;22(6):920–33. doi: 10.1261/rna.054916.115 (PMC4878617; doi:10.1261/rna.054916.115)
Supplement: Supplemental Material [file supp_054916.115_Supplemental_Material.pdf]

Supplementary Information for

Using in-cell SHAPE-Seq and simulations to probe structure-function design principles of RNA transcriptional regulators

Melissa K. Takahashi[1,3], Kyle E. Watters[1], Paul M. Gasper[2], Timothy R. Abbott[1,4], Paul D. Carlson[1], Alan A. Chen[2], Julius B. Lucks[1,*]

[1] School of Chemical and Biomolecular Engineering, Cornell University, Ithaca, NY, 14850, USA
[2] Department of Chemistry and RNA Institute, University at Albany, Albany, NY, 12222, USA
[3] Present address: Institute for Medical Engineering and Science, Massachusetts Institute of Technology, Cambridge, MA 02139, USA
[4] Present address: Department of Bioengineering, Stanford University, Palo Alto, CA, 94305, USA

* To whom correspondence should be addressed. Tel: 1-607-379-0865; Fax: 1-607-255-9166; Email: jblucks@cornell.edu

Table of contents:

	Description	Page
Supplementary Methods	Simulation details	2
Supplementary Table S1	Plasmids used in this study	4
Supplementary Table S2	Important DNA sequences	7
Supplementary Table S3	Attenuator sequences	10
Supplementary Table S4	Antisense sequences	13
Supplementary Table S5	Oligonucleotides used for in-cell SHAPE-Seq	14
Supplementary Table S6	RMDB data deposition table	15
Supplementary Table S7	Thermodynamic folding analysis of ON and OFF structures	16
Supplementary Figure S1	Plasmid architecture	17
Supplementary Figure S2	Full comparison of in-cell SHAPE-Seq reactivity spectra for Fusions 1-3	18
Supplementary Figure S3	In-cell SHAPE-Seq reactivity comparison for Fusions 1-3 antisense	20
Supplementary Figure S4	Full comparison of in-cell SHAPE-Seq reactivity spectra for Fusions 10 and 4	21
Supplementary Figure S5	Comparison of interior loop closures to non-functional fusions	22
Supplementary Figure S6	Mutations to the 3’ side of Fusion 3 interior loops	23
Supplementary Figure S7	Mutations to the 3’ side of Fusion 4 interior loop	24
Supplementary Figure S8	Percent base pair occupancies from molecular dynamics simulations	25
Supplementary Figure S9	Simulation cumulative average base pair occupancy	26
Supplementary Figure S10	Representative simulation structures	27
Supplementary Figure S11	Full in-cell SHAPE-Seq reactivity profiles for natural translational regulator hairpins	28
Supplementary Figure S12	Using NUPACK to design chimeric attenuators with defined interior loops	29
Supplementary Figure S13	Full in-cell SHAPE-Seq reactivity profiles for NUPACK fusions compared to Fusion 4	30
Supplementary Note	Code used to design *in silico* attenuators using the NUPACK webservers	31
Supplementary Movie 1-2	Movie of 2 ns segments from REMD simulations of Fusion 3 and Fusion 3 L2(GU-CA)	32

Supplementary Methods

Simulation details

All-atom, replica exchange molecular dynamics (REMD) were performed for Fusion 3 and Fusion 3 L2(GU-CA) sense strand, hairpins (nucleotides 14-61) using the GROMACS software package version 5.0.4 (Pronk et al. 2013). The Amber-99 force field (Wang et al. 2000) ported to GROMACS by Sorin and Pande (Sorin and Pande 2005) was used with modifications for nucleic acids introduced by Chen and Garcia (Chen and García 2013). Further improvements to the nucleic acid torsion and base-pairing potentials calibrated against ultrasonic absorption (Nishikawa et al. 2000) and NMR relaxation dispersion (Rinnenthal et al. 2010) were incorporated. A total of 13209 and 13186 explicit water molecules were added to the Fusion 3 and Fusion 3 L2(GU-CA) systems respectively with the TIP3P model (Jorgensen et al. 1983). Additionally 86 Na^+ and 39 Cl^- ions were added to each system to neutralize the net charge and bring the salt concentration to 0.15 M. Ions were modeled using parameters by Åqvist (Aaqvist 1990) following the approach by Chen and Pappu (Chen and Pappu 2007) to eliminate spurious ion-pairing artifacts.

Each RNA was centered in a 6.0 x 6.0 x 12.0 Å box and aligned to the principle axis.
The box size was chosen based on maintaining a minimum distance of 10 Å between periodic images in the conformational space explored during preliminary, long, high-temperature simulations. A rough alignment with the principle axis was maintained by the application of 3 Å, flat-bottomed, cylindrical restraints with weak, 100 kJ/mol force constant, harmonic edges to the C5' residues at the base of the stem and a 5 Å, flat-bottomed, spherical restraint with weak, 50 kJ/mol force constant, harmonic edges to the C3' residue at the center of the loop. Long-range electrostatic interactions were treated using the particle mesh Ewald approach (Cheatham et al. 1995).

Initial, all-atom RNA structures were generated by the MC-Sym package (Parisien and Major 2008) using secondary structures generated by RNAstructure (Reuter and Mathews 2010) as input. A steepest decent energy minimization was performed until the maximum force was less than 100.0 kJ/mol/nm. A 100 ps, constant volume simulation was used to equilibrate the temperature to 300 K and was followed by a 100 ps, constant pressure equilibration at 1 bar. Production simulations were performed for 130 ns with conformational sampling by replica exchange. Constant temperature was maintained for each replica using a modified Berendsen thermostat with a tau-t of 0.1 ps. A 2 fs time step was used and snapshots were saved every 2 ps. The first 30 ns were considered equilibration based on analysis of cumulative average base pair occupancy (Supplementary Figure S9).

A preliminary REMD temperature schedule was generated using the temperature predictor algorithm by Patriksson and van der Spoel (Patriksson and van der Spoel 2008). A 1 ns REMD run was used to optimize the temperature schedule by calculation of the rate of acceptance using Gaussian energy distributions as implemented by Garcia and Paschek (García et al. 2006). The resulting schedule comprised 66 replicates ranging from 290.00 K to 435.10 K. The upper temperature limit was selected to permit significant melting of loop and fusion region while leave the stem relatively intact. Exchange rates of 25% were obtained with swaps attempted every 2 ps.

Supplementary Table S1: Plasmids used in this study. Abbreviations are as follows. TrrnB = rrnB terminator, CmR = chloramphenicol resistance, AmpR = ampicillin resistance, SFGFP = super folder green fluorescent protein, t500 = T500 terminator, ECK120051404 = ECK120051404 terminator (Chen et al. 2013).

Plasmid number	Plasmid architecture	Name	Figures
JBL001	TrrnB – backbone (p15A origin/CmR)	No attenuator control	N/A
JBL002	J23119 – TrrnB – backbone (ColE1 origin/AmpR)	No antisense control	N/A
JBL006	J23119 – pT181 attenuator – SFGFP – TrrnB – backbone (p15A origin/CmR)	pT181	2
JBL1521	J23119 – pT181 antisense – g – ECK120051404 – t500 – backbone (ColE1 origin/AmpR)	pT181 antisense	2
JBL1815	J23119 – Fusion 1 attenuator – TrrnB – backbone (p15A origin/CmR)	Fusion 1	2
JBL1954	J23119 – Fusion 1 antisense – g – ECK120051404 – t500 – backbone (ColE1 origin/AmpR)	Fusion 1 antisense	2, S2
JBL1017	J23119 – Fusion 2 attenuator – SFGFP – TrrnB – backbone (p15A origin/CmR)	Fusion 2	2
JBL1920	J23119 – Fusion 2 antisense – g – ECK120051404 – t500 – backbone (ColE1 origin/AmpR)	Fusion 2 antisense	2, S2
JBL1039	J23119 – Fusion 3 attenuator – SFGFP – TrrnB – backbone (p15A origin/CmR)	Fusion 3	2, 3
JBL1921	J23119 – Fusion 3 antisense – g – ECK120051404 – t500 – backbone (ColE1 origin/AmpR)	Fusion 3 antisense	2, 3, S2
JBL1037	J23119 – Fusion 10 attenuator – SFGFP – TrrnB – backbone (p15A origin/CmR)	Fusion 10	2
JBL1918	J23119 – Fusion 10 antisense – g – ECK120051404 – t500 – backbone (ColE1 origin/AmpR)	Fusion 10 antisense	2
JBL1126	J23119 – Fusion 4 attenuator – SFGFP – TrrnB – backbone (p15A origin/CmR)	Fusion 4	2, 3
JBL1919	J23119 – Fusion 4 antisense – g – ECK120051404 – t500 – backbone (ColE1 origin/AmpR)	Fusion 4 antisense	2, 3
JBL1932	J23119 – Fusion 3 L1(UU-AA) attenuator – SFGFP – TrrnB – backbone (p15A origin/CmR)	Fusion 3 L1(UU-AA)	3
JBL1977	J23119 – Fusion 3 L1(UU-AA) antisense – g – ECK120051404 – t500 – backbone (ColE1 origin/AmpR)	Fusion 3 L1(UU-AA) antisense	3
JBL1927	J23119 – Fusion 3 L1(AA-UU) attenuator – SFGFP – TrrnB – backbone (p15A origin/CmR)	Fusion 3 L1(AA-UU)	S6
JBL1978	J23119 – Fusion 3 L1(AA-UU) antisense – g – ECK120051404 – t500 – backbone (ColE1 origin/AmpR)	Fusion 3 L1(AA-UU) antisense	S6
JBL1933	J23119 – Fusion 3 L2(UC-AG) attenuator – SFGFP – TrrnB – backbone (p15A origin/CmR)	Fusion 3 L2(UC-AG)	S6
JBL1979	J23119 – Fusion 3 L2(UC-AG) antisense – g – ECK120051404 – t500 – backbone (ColE1 origin/AmpR)	Fusion 3 L2(UC-AG) antisense	S6
JBL1928	J23119 – Fusion 3 L2(GU-CA) attenuator – SFGFP – TrrnB – backbone (p15A origin/CmR)	Fusion 3 L2(GU-CA)	3

JBL1980	J23119 – Fusion 3 L2(GU-CA) antisense – g – ECK120051404 – t500 – backbone (ColE1 origin/AmpR)	Fusion 3 L2(GU-CA) antisense	3
JBL1948	J23119 – Fusion 4 L(UG-AC) attenuator – SFGFP – TrrnB – backbone (p15A origin/CmR)	Fusion 4 L(UG-AC)	3
JBL1982	J23119 – Fusion 4 L(UG-AC) antisense – g – ECK120051404 – t500 – backbone (ColE1 origin/AmpR)	Fusion 4 L(UG-AC) antisense	3
JBL1949	J23119 – Fusion 4 L(AC-UG) attenuator – SFGFP – TrrnB – backbone (p15A origin/CmR)	Fusion 4 L(UG-AC)	S7
JBL1962	J23119 – Fusion 4 L(AC-UG) antisense – g – ECK120051404 – t500 – backbone (ColE1 origin/AmpR)	Fusion 4 L(UG-AC) antisense	S7
JBL5232	J23119 – NUPACK Fusion 1 attenuator – SFGFP – TrrnB – backbone (p15A origin/CmR)	NP Fusion 1	6
JBL5233	J23119 – NUPACK Fusion 1 antisense – TrrnB – backbone (ColE1 origin/AmpR)	NP Fusion 1 antisense	6
JBL5234	J23119 – NUPACK Fusion 2 attenuator – SFGFP – TrrnB – backbone (p15A origin/CmR)	NP Fusion 2	6
JBL5235	J23119 – NUPACK Fusion 2 antisense – TrrnB – backbone (ColE1 origin/AmpR)	NP Fusion 2 antisense	6
JBL5236	J23119 – NUPACK Fusion 1 attenuator (112) – g – ECK120051404 – t500 – backbone (p15A origin/CmR)	NP Fusion 1 (SHAPE)	6, S13
JBL5237	J23119 – NUPACK Fusion 2 attenuator (112) – g – ECK120051404 – t500 – backbone (p15A origin/CmR)	NP Fusion 2 (SHAPE)	6, S13
JBL1941	J23119 – Fusion 1 attenuator (112) – g – ECK120051404 – t500 – backbone (p15A origin/CmR)	Fusion 1 (SHAPE)	2, S2
JBL1916	J23119 – Fusion 2 attenuator (112) – g – ECK120051404 – t500 – backbone (p15A origin/CmR)	Fusion 2 (SHAPE)	2, S2, S5
JBL1917	J23119 – Fusion 3 attenuator (112) – g – ECK120051404 – t500 – backbone (p15A origin/CmR)	Fusion 3 (SHAPE)	2, 3, 4, 5, S2, S6, S8
JBL1914	J23119 – Fusion 10 attenuator (112) – g – ECK120051404 – t500 – backbone (p15A origin/CmR)	Fusion 10 (SHAPE)	2, S4, S5
JBL1915	J23119 – Fusion 4 attenuator (112) – g – ECK120051404 – t500 – backbone (p15A origin/CmR)	Fusion 4 (SHAPE)	2, 3, 5, S4
JBL1522	J23119 – pT181 attenuator (112) – g – ECK120051404 – t500 – backbone (p15A origin/CmR)	pT181 (SHAPE)	1
JBL1974	J23119 – Fusion 3 L1(UU-AA) attenuator (112) – g – ECK120051404 – t500 – backbone (p15A origin/CmR)	Fusion 3 L1(UU-AA) (SHAPE)	3
JBL1975	J23119 – Fusion 3 L1(AA-UU) attenuator (112) – g – ECK120051404 – t500 – backbone (p15A origin/CmR)	Fusion 3 L1(AA-UU) (SHAPE)	S6
JBL1984	J23119 – Fusion 3 L2(GU-CA) attenuator (112) – g – ECK120051404 – t500 – backbone (p15A origin/CmR)	Fusion 3 L2(GU-CA) (SHAPE)	3, 4, S5, S8
JBL1976	J23119 – Fusion 3 L2(UC-AG) attenuator (112) – g – ECK120051404 – t500 – backbone (p15A origin/CmR)	Fusion 3 L2(UC-AG) (SHAPE)	S6
JBL1996	J23119 – Fusion 4 L(UG-AC) attenuator (112) – g – ECK120051404 – t500 – backbone (p15A origin/CmR)	Fusion 4 L(UG-AC) (SHAPE)	3, S5
JBL1997	J23119 – Fusion 4 L(AC-UG) attenuator (112) – g – ECK120051404 – t500 – backbone (p15A origin/CmR)	Fusion 4 L(AC-UG) (SHAPE)	S7

JBL3273	J23119 – R1 hairpin1 – g – ECK120051404 – t500 – backbone (p15A origin/CmR)	R1 (SHAPE)	5, S11
JBL3317	J23119 – pMU720 hairpin1 – g – ECK120051404 – t500 – backbone (p15A origin/CmR)	pMU720 (SHAPE)	5, S11
JBL3286	J23119 – NUPACK Fusion 3 attenuator – SFGFP – TrrnB – backbone (p15A origin/CmR)	NP Fusion 3	S12
JBL3288	J23119 – NUPACK Fusion 4 attenuator – SFGFP – TrrnB – backbone (p15A origin/CmR)	NP Fusion 4	S12
JBL3287	J23119 – NUPACK Fusion 3 antisense – TrrnB – backbone (ColE1 origin/AmpR)	NP Fusion 3 antisense	S12
JBL3289	J23119 – NUPACK Fusion 4 antisense – TrrnB – backbone (ColE1 origin/AmpR)	NP Fusion 3 antisense	S12
JBL3309	J23119 – NUPACK Fusion 3 attenuator (112) – g – ECK120051404 – t500 – backbone (p15A origin/CmR)	NP Fusion 3 (SHAPE)	S12
JBL3313	J23119 – NUPACK Fusion 4 attenuator (112) – g – ECK120051404 – t500 – backbone (p15A origin/CmR)	NP Fusion 4 (SHAPE)	S12

Supplementary Table S2: Important DNA sequences. Abbreviations are as follows. TrrnB = rrnB terminator, CmR = chloramphenicol resistance, AmpR = ampicillin resistance, SFGFP = super folder green fluorescent protein, t500 = T500 terminator, ECK120051404 = ECK120051404 terminator (Chen et al. 2013).

Name	Sequence
Backbone (p15A origin/CmR)<br><br>CmR – p15A origin	GGATCCTTACTCGAGTCTAGACTGCAGTTGATCGGGCACGTAAGAGGTTCCA<br>ACTTTCACCATAATGAAATAAGATCACTACCGGGCGTATTTTTTGAGTTATCGA<br>GATTTTCAGGAGCTAAGGAAGCTAAAATGGAGAAAAAAATCACTGGATATACC<br>ACCGTTGATATATCCCAATGGCATCGTAAAGAACATTTTGAGGCATTTCAGTC<br>AGTTGCTCAATGTACCTATAACCAGACCGTTCAGCTGGATATTACGGCCTTTT<br>TAAAGACCGTAAAGAAAAATAAGCACAAGTTTTATCCGGCCTTTATTCACATTC<br>TTGCCCGCCTGATGAATGCTCATCCGGAATTTCGTATGGCAATGAAAGACGGT<br>GAGCTGGTGATATGGGATAGTGTTCACCCTTGTTACACCGTTTTCCATGAGCA<br>AACTGAAACGTTTTCATCGCTCTGGAGTGAATACCACGACGATTTCCGGCAGT<br>TTCTACACATATATTCGCAAGATGTGGCGTGTTACGGTGAAAACCTGGCCTAT<br>TTCCCTAAAGGGTTTATTGAGAATATGTTTTTCGTCTCAGCCAATCCCTGGGT<br>GAGTTTCACCAGTTTTGATTTAAACGTGGCCAATATGGACAACTTCTTCGCCC<br>CCGTTTTCACCATGGGCAAATATTATACGCAAGGCGACAAGGTGCTGATGCC<br>GCTGGCGATTCAGGTTCATCATGCCGTTTGTGATGGCTTCCATGTCGGCAGA<br>ATGCTTAATGAATTACAACAGTACTGCGATGAGTGGCAGGGCGGGGCGTAAT<br>TTGATATCGAGCTCGCTTGGACTCCTGTTGATAGATCCAGTAATGACCTCAGA<br>ACTCCATCTGGATTTGTTCAGAACGCTCGGTTGCCGCCGGGCGTTTTTTATTG<br>GTGAGAATCCAAGCCTCCGATCAACGTCTCATTTTCGCCAAAAGTTGGCCCAG<br>GGCTTCCCGGTATCAACAGGGACACCAGGATTTATTTATTCTGCGAAGTGATC<br>TTCCGTCACAGGTATTTATTCGGCGCAAAGTGCGTCGGGTGATGCTGCCAAC<br>TTACTGATTTAGTGTATGATGGTGTTTTTGAGGTGCTCCAGTGGCTTCTGTTTC<br>TATCAGCTGTCCCTCCTGTTCAGCTACTGACGGGGTGGTGCGTAACGGCAAA<br>AGCACCGCCGGACATCAGCGCTAGCGGAGTGTATACTGGCTTACTATGTTGG<br>CACTGATGAGGGTGTCAGTGAAGTGCTTCATGTGGCAGGAGAAAAAAGGCTG<br>CACCGGTGCGTCAGCAGAATATGTGATACAGGATATATTCCGCTTCCTCGCTC<br>ACTGACTCGCTACGCTCGGTCGTTCGACTGCGGCGAGCGGAAATGGCTTACG<br>AACGGGGCGGAGATTTCCTGGAAGATGCCAGGAAGATACTTAACAGGGAAGT<br>GAGAGGGCCGCGGCAAAGCCGTTTTTCCATAGGCTCCGCCCCCCTGACAAG<br>CATCACGAAATCTGACGCTCAAATCAGTGGTGGCGAAACCCGACAGGACTAT<br>AAAGATACCAGGCGTTTCCCCCTGGCGGCTCCCTCGTGCGCTCTCCTGTTCC<br>TGCCTTTCGGTTTACCGGTGTCATTCCGCTGTTATGGCCGCGTTTGTCTCATT<br>CCACGCCTGACACTCAGTTCCGGGTAGGCAGTTCGCTCCAAGCTGGACTGTA<br>TGCACGAACCCCCCGTTCAGTCCGACCGCTGCGCCTTATCCGGTAACTATCG<br>TCTTGAGTCCAACCCGGAAAGACATGCAAAAGCACCACTGGCAGCAGCCACT<br>GGTAATTGATTTAGAGGAGTTAGTCTTGAAGTCATGCGCCGGTTAAGGCTAAA<br>CTGAAAGGACAAGTTTTGGTGACTGCGCTCCTCCAAGCCAGTTACCTCGGTT<br>CAAAGAGTTGGTAGCTCAGAGAACCTTCGAAAAACCGCCCTGCAAGGCGGTT<br>TTTTCGTTTTCAGAGCAAGAGATTACGCGCAGACCAAAACGATCTCAAGAAGA<br>TCATCTTATTAATCAGATAAAATATTT
Backbone (ColE1 origin/AmpR)<br><br>ColE1 origin – AmpR	GGATCCTTACTCGAGTCTAGACTGCAGGCTTCCTCGCTCACTGACTCGCTGC<br>GCTCGGTCGTTCGGCTGCGGCGAGCGGTATCAGCTCACTCAAAGGCGGTAA<br>TACGGTTATCCACAGAATCAGGGGATAACGCAGGAAAGAACATGTGAGCAAA<br>AGGCCAGCAAAAGGCCAGGAACCGTAAAAAGGCCGCGTTGCTGGCGTTTTTC<br>CACAGGCTCCGCCCCCCTGACGAGCATCACAAAAATCGACGCTCAAGTCAGA<br>GGTGGCGAAACCCGACAGGACTATAAAGATACCAGGCGTTTCCCCCTGGAAG<br>CTCCCTCGTGCGCTCTCCTGTTCCGACCCTGCCGCTTACCGGATACCTGTCC<br>GCCTTTCTCCCTTCGGGAAGCGTGGCGCTTTCTCATAGCTCACGCTGTAGGT<br>ATCTCAGTTCGGTGTAGGTCGTTCGCTCCAAGCTGGGCTGTGTGCACGAACC<br>CCCCGTTCAGCCCGACCGCTGCGCCTTATCCGGTAACTATCGTCTTGAGTCC

	AACCCGGTAAGACACGACTTATCGCCACTGGCAGCAGCCACTGGTAACAGGA<br>TTAGCAGAGCGAGGTATGTAGGCGGTGCTACAGAGTTCTTGAAGTGGTGGCC<br>TAACTACGGCTACACTAGAAGAACAGTATTTGGTATCTGCGCTCTGCTGAAGC<br>CAGTTACCTTCGGAAAAAGAGTTGGTAGCTCTTGATCCGGCAAACAAACCACC<br>GCTGGTAGCGGTGGTTTTTTTGTTTGCAAGCAGCAGATTACGCGCAGAAAAAA<br>AGGATCTCAAGAAGATCCTTTGATCTTTTCTACGGGGTCTGACGCTCAGTGGA<br>ACGAAAACTCACGTTAAGGGATTTTGGTCATGAGATTATCAAAAAGGATCTTC<br>ACCTAGATCCTTTTAAATTAAAAATGAAGTTTTAAATCAATCTAAAGTATATATG<br>AGTAAACTTGGTCTGACAGTTACCAATGCTTAATCAGTGAGGCACCTATCTCA<br>GCGATCTGTCTATTTCGTTCATCCATAGTTGCCTGACTCCCCGTCGTGTAGAT<br>AACTACGATACGGGAGGGCTTACCATCTGGCCCCAGTGCTGCAATGATACCG<br>CGAGACCCACGCTCACCGGCTCCAGATTTATCAGCAATAAACCAGCCAGCCG<br>GAAGGGCCGAGCGCAGAAGTGGTCCTGCAACTTTATCCGCCTCCATCCAGTC<br>TATTAATTGTTGCCGGGAAGCTAGAGTAAGTAGTTCGCCAGTTAATAGTTTGC<br>GCAACGTTGTTGCCATTGCTACAGGCATCGTGGTGTCACGCTCGTCGTTTGG<br>TATGGCTTCATTCAGCTCCGGTTCCCAACGATCAAGGCGAGTTACATGATCCC<br>CCATGTTGTGCAAAAAAGCGGTTAGCTCCTTCGGTCCTCCGATCGTTGTCAGA<br>AGTAAGTTGGCCGCAGTGTTATCACTCATGGTTATGGCAGCACTGCATAATTC<br>TCTTACTGTCATGCCATCCGTAAGATGCTTTTCTGTGACTGGTGAGTACTCAA<br>CCAAGTCATTCTGAGAATAGTGTATGCGGCGACCGAGTTGCTCTTGCCCGGC<br>GTCAATACGGGATAATACCGCGCCACATAGCAGAACTTTAAAAGTGCTCATCA<br>TTGGAAAACGTTCTTCGGGGCGAAAACTCTCAAGGATCTTACCGCTGTTGAGA<br>TCCAGTTCGATGTAACCCACTCGTGCACCCAACTGATCTTCAGCATCTTTTAC<br>TTTCACCAGCGTTTCTGGGTGAGCAAAAACAGGAAGGCAAAATGCCGCAAAA<br>AAGGGAATAAGGGCGACACGGAAATGTTGAATACTCATACTCTTCCTTTTTCA<br>ATATTATTGAAGCATTTATCAGGGTTATTGTCTCATGAGCGGATACATATTTGA<br>ATGTATTTAGAAAAATAAACAAATAGGGGTTCCGCGCACATTTCCCCGAAAAG<br>TGCCACCTGACGTCTAAGAAACCATTATTATCATGACATTAACCTATAAAAATA<br>GGCGTATCACGAGGCAGAATTTCAGATAAAAAAAATCCTTAGCTTTCGCTAAG<br>GATGATTTCTG
Example attenuator-SFGFP construct (EcoRI - J23119 – pT181 attenuator – RBS – SFGFP – TrrnB)	GAATTCTAAAGATCTTTGACAGCTAGCTCAGTCCTAGGTATAATACTAGTAACA<br>AAATAAAAAGGAGTCGCTCACGCCCTGACCAAAGTTTGTGAACGACATCATTC<br>AAAGAAAAAAACACTGAGTTGTTTTTATAATCTTGTATATTTAGATATTAAACGA<br>TATTTAAATATACATAAAGATATATATTTGGGTGAGCGATTCCTTAAACGAAATT<br>GAGATTAAGGAGTCGCTCTTTTTTATGTATAAAAACAATCATGCAAATCATTCA<br>AATCATTTGGAAAATCACGATTTAGACAATTTTTCTAAAACCGGCTACTCTAAT<br>AGCCGGTTGTAAGGATCTAGGAGGAAGGATCTATGAGCAAAGGAGAAGAACT<br>TTTCACTGGAGTTGTCCCAATTCTTGTTGAATTAGATGGTGATGTTAATGGGCA<br>CAAATTTTCTGTCCGTGGAGAGGGTGAAGGTGATGCTACAAACGGAAAACTC<br>ACCCTTAAATTTATTTGCACTACTGGAAAACTACCTGTTCCGTGGCCAACACTT<br>GTCACTACTCTGACCTATGGTGTTCAATGCTTTTCCCGTTATCCGGATCACAT<br>GAAACGGCATGACTTTTTCAAGAGTGCCATGCCCGAAGGTTATGTACAGGAA<br>CGCACTATATCTTTCAAAGATGACGGGACCTACAAGACGCGTGCTGAAGTCAA<br>GTTTGAAGGTGATACCCTTGTTAATCGTATCGAGTTAAAGGGTATTGATTTTAA<br>AGAAGATGGAAACATTCTTGGACACAAACTCGAGTACAACTTTAACTCACACA<br>ATGTATACATCACGGCAGACAAACAAAAGAATGGAATCAAAGCTAACTTCAAA<br>ATTCGCCACAACGTTGAAGATGGTTCCGTTCAACTAGCAGACCATTATCAACA<br>AAATACTCCAATTGGCGATGGCCCTGTCCTTTTACCAGACAACCATTACCTGT<br>CGACACAATCTGTCCTTTCGAAAGATCCCAACGAAAAGCGTGACCACATGGTC<br>CTTCTTGAGTTTGTAACTGCTGCTGGGATTACACATGGCATGGATGAGCTCTA<br>CAAATAAGGATCTGAAGCTTGGGCCCGAACAAAAACTCATCTCAGAAGAGGAT<br>CTGAATAGCGCCGTCGACCATCATCATCATCATCATTGAGTTTAAACGGTCTC<br>CAGCTTGGCTGTTTTGGCGGATGAGAGAAGATTTTCAGCCTGATACAGATTAA<br>ATCAGAACGCAGAAGCGGTCTGATAAAACAGAATTTGCCTGGCGGCAGTAGC<br>GCGGTGGTCCCACCTGACCCCATGCCGAACTCAGAAGTGAAACGCCGTAGC<br>GCCGATGGTAGTGTGGGGTCTCCCCATGCGAGAGTAGGGAACTGCCAGGCA

	TCAAATAAAACGAAAGGCTCAGTCGAAAGACTGGGCCTTTCGTTTTATCTGTT GTTTGTCGGTGAACT
Example antisense construct<br><br>(EcoRI – J23119 – pT181 antisense – g-ECK120051404 – t500)	GAATTCTAAAGATCTTTGACAGCTAGCTCAGTCCTAGGTATAATACTAGTATAC AAGATTATAAAAACAACTCAGTGTTTTTTTCTTTGAATGATGTCGTTCACAAACT TTGGTCAGGGCGTGAGCGACTCCTTTTTATTTGCCTCTACCTGCTTCGGCCGA TAAAGCCGACGATAATACTCCCAAAGCCCGCCGAAAGGCGGGCTTTTTTTT
Example attenuator SHAPE construct<br><br>(EcoRI – J23119 – pT181 attenuator (112) – g – ECK120051404 – t500)	GAATTCTAAAGATCTTTGACAGCTAGCTCAGTCCTAGGTATAATACTAGTAACA AAATAAAAAGGAGTCGCTCACGCCCTGACCAAAGTTTGTGAACGACATCATTC AAAGAAAAAAACACTGAGTTGTTTTTATAATCTTGTATATTTAGATATTAAACGA GCCTCTACCTGCTTCGGCCGATAAAGCCGACGATAATACTCCCAAAGCCCGC CGAAAGGCGGGCTTTTTTTT

Supplementary Table S3: Attenuator sequences.

Name	Sequence
pT181	AACAAAATAAAAAGGAGTCGCTCACGCCCTGACCAAAGTTTGTGAACGACATC<br>ATTCAAAGAAAAAAACACTGAGTTGTTTTTATAATCTTGTATATTTAGATATTAA<br>ACGATATTTAAATATACATAAAGATATATATTTGGGTGAGCGATTCCTTAAACG<br>AAATTGAGATTAAGGAGTCGCTCTTTTTTATGTATAAAAACAATCATGCAAATC<br>ATTCAAATCATTTGGAAAATCACGATTTAGACAATTTTTCTAAAACCGGCTACT<br>CTAATAGCCGGTTGTAA
Fusion 1	AACAAAATAAAAAGGAGTCGCTCACGCTTGGCGGTGTGAACGACATCATTCAA<br>AGAAAAAAACACTGAGTTGTTTTTATAATCTTGTATATTTAGATATTAAACGATA<br>TTTAAATATACATAAAGATATATATTTGGGTGAGCGATTCCTTAAACGAAATTG<br>AGATTAAGGAGTCGCTCTTTTTTATGTATAAAAACAATCATGCAAATCATTCAA<br>ATCATTTGGAAAATCACGATTTAGACAATTTTTCTAAAACCGGCTACTCTAATA<br>GCCGGTTGTAA
Fusion 2	AACAAAATAAAAAGGAGTCGCTCACGGAACTTGGCGGAACTGTGAACGACAT<br>CATTCAAAGAAAAAAACACTGAGTTGTTTTTATAATCTTGTATATTTAGATATTA<br>AACGATATTTAAATATACATAAAGATATATATTTGGGTGAGCGATTCCTTAAAC<br>GAAATTGAGATTAAGGAGTCGCTCTTTTTTATGTATAAAAACAATCATGCAAAT<br>CATTCAAATCATTTGGAAAATCACGATTTAGACAATTTTTCTAAAACCGGCTAC<br>TCTAATAGCCGGTTGTAA
Fusion 3	AACAAAATAAAAAGGAGTCGCTCACGCCTCGAACTTGGCGGAACGCAGTGTG<br>AACGACATCATTCAAAGAAAAAAACACTGAGTTGTTTTTATAATCTTGTATATTT<br>AGATATTAAACGATATTTAAATATACATAAAGATATATATTTGGGTGAGCGATT<br>CCTTAAACGAAATTGAGATTAAGGAGTCGCTCTTTTTTATGTATAAAAACAATC<br>ATGCAAATCATTCAAATCATTTGGAAAATCACGATTTAGACAATTTTTCTAAAAC<br>CGGCTACTCTAATAGCCGGTTGTAA
Fusion 10	AACAAAATAAAAAGGAGTCGCTCACGCTTTGGCGAGTGTGAACGACATCATTC<br>AAAGAAAAAAACACTGAGTTGTTTTTATAATCTTGTATATTTAGATATTAAACGA<br>TATTTAAATATACATAAAGATATATATTTGGGTGAGCGATTCCTTAAACGAAATT<br>GAGATTAAGGAGTCGCTCTTTTTTATGTATAAAAACAATCATGCAAATCATTCA<br>AATCATTTGGAAAATCACGATTTAGACAATTTTTCTAAAACCGGCTACTCTAAT<br>AGCCGGTTGTAA
Fusion 4	AACAAAATAAAAAGGAGTCGCTCACGTTCAACTTTGGCGAGTACGATGTGAAC<br>GACATCATTCAAAGAAAAAAACACTGAGTTGTTTTTATAATCTTGTATATTTAGA<br>TATTAAACGATATTTAAATATACATAAAGATATATATTTGGGTGAGCGATTCCTT<br>AAACGAAATTGAGATTAAGGAGTCGCTCTTTTTTATGTATAAAAACAATCATGC<br>AAATCATTCAAATCATTTGGAAAATCACGATTTAGACAATTTTTCTAAAACCGG<br>CTACTCTAATAGCCGGTTGTAA
Fusion 3<br>L1(UU-AA)	AACAAAATAAAAAGGAGTCGCTCACGCCTCGTTCTTGGCGGAACGCAGTGTG<br>AACGACATCATTCAAAGAAAAAAACACTGAGTTGTTTTTATAATCTTGTATATTT<br>AGATATTAAACGATATTTAAATATACATAAAGATATATATTTGGGTGAGCGATT<br>CCTTAAACGAAATTGAGATTAAGGAGTCGCTCTTTTTTATGTATAAAAACAATC<br>ATGCAAATCATTCAAATCATTTGGAAAATCACGATTTAGACAATTTTTCTAAAAC<br>CGGCTACTCTAATAGCCGGTTGTAA
Fusion 3<br>L1(AA-UU)	AACAAAATAAAAAGGAGTCGCTCACGCCTCGAACTTGGCGGTTCGCAGTGTG<br>AACGACATCATTCAAAGAAAAAAACACTGAGTTGTTTTTATAATCTTGTATATTT<br>AGATATTAAACGATATTTAAATATACATAAAGATATATATTTGGGTGAGCGATT<br>CCTTAAACGAAATTGAGATTAAGGAGTCGCTCTTTTTTATGTATAAAAACAATC<br>ATGCAAATCATTCAAATCATTTGGAAAATCACGATTTAGACAATTTTTCTAAAAC<br>CGGCTACTCTAATAGCCGGTTGTAA
Fusion 3<br>L2(UC-AG)	AACAAAATAAAAAGGAGTCGCTCACGCCTCGAACTTGGCGGAACGAGGTGTG<br>AACGACATCATTCAAAGAAAAAAACACTGAGTTGTTTTTATAATCTTGTATATTT<br>AGATATTAAACGATATTTAAATATACATAAAGATATATATTTGGGTGAGCGATT<br>CCTTAAACGAAATTGAGATTAAGGAGTCGCTCTTTTTTATGTATAAAAACAATC

	ATGCAAATCATTCAAATCATTTGGAAAATCACGATTTAGACAATTTTTCTAAAAC CGGCTACTCTAATAGCCGGTTGTAA
Fusion 3 L2(GU-CA)	AACAAAATAAAAAGGAGTCGCTCACGCTGCGAACTTGGCGGAACGCAGTGTG AACGACATCATTCAAAGAAAAAAACACTGAGTTGTTTTTATAATCTTGTATATTT AGATATTAAACGATATTTAAATATACATAAAGATATATATTTGGGTGAGCGATT CCTTAAACGAAATTGAGATTAAGGAGTCGCTCTTTTTTATGTATAAAAACAATC ATGCAAATCATTCAAATCATTTGGAAAATCACGATTTAGACAATTTTTCTAAAAC CGGCTACTCTAATAGCCGGTTGTAA
Fusion 4 L(UG-AC)	AACAAAATAAAAAGGAGTCGCTCACGTTGTACTTTGGCGAGTACGATGTGAAC GACATCATTCAAAGAAAAAAACACTGAGTTGTTTTTATAATCTTGTATATTTAGA TATTAAACGATATTTAAATATACATAAAGATATATATTTGGGTGAGCGATTCCTT AAACGAAATTGAGATTAAGGAGTCGCTCTTTTTTATGTATAAAAACAATCATGC AAATCATTCAAATCATTTGGAAAATCACGATTTAGACAATTTTTCTAAAACCGG CTACTCTAATAGCCGGTTGTAA
Fusion 4 L(AC-UG)	AACAAAATAAAAAGGAGTCGCTCACGTTCAACTTTGGCGAGTTGGATGTGAAC GACATCATTCAAAGAAAAAAACACTGAGTTGTTTTTATAATCTTGTATATTTAGA TATTAAACGATATTTAAATATACATAAAGATATATATTTGGGTGAGCGATTCCTT AAACGAAATTGAGATTAAGGAGTCGCTCTTTTTTATGTATAAAAACAATCATGC AAATCATTCAAATCATTTGGAAAATCACGATTTAGACAATTTTTCTAAAACCGG CTACTCTAATAGCCGGTTGTAA
NP Fusion 1	AACAAAATAAAAAGGAGTCGCTCACGGAGGCCTTGGCGGGAGTCTGTGAACG ACATCATTCAAAGAAAAAAACACTGAGTTGTTTTTATAATCTTGTATATTTAGAT ATTAAACGATATTTAAATATACATAAAGATATATATTTGGGTGAGCGATTCCTTA AACGAAATTGAGATTAAGGAGTCGCTCTTTTTTATGTATAAAAACAATCATGCA AATCATTCAAATCATTTGGAAAATCACGATTTAGACAATTTTTCTAAAACCGGC TACTCTAATAGCCGGTTGTAA
NP Fusion 2	AACAAAATAAAAAGGAGTCGCTCACGGGGGTGTTGGCGCAGACCTGTGAACG ACATCATTCAAAGAAAAAAACACTGAGTTGTTTTTATAATCTTGTATATTTAGAT ATTAAACGATATTTAAATATACATAAAGATATATATTTGGGTGAGCGATTCCTTA AACGAAATTGAGATTAAGGAGTCGCTCTTTTTTATGTATAAAAACAATCATGCA AATCATTCAAATCATTTGGAAAATCACGATTTAGACAATTTTTCTAAAACCGGC TACTCTAATAGCCGGTTGTAA
NP Fusion 3	AACAAAATAAAAAGGAGTCGCTCACGAAGACCGCCCTCCCTGTGAACGACAT CATTCAAAGAAAAAAACACTGAGTTGTTTTTATAATCTTGTATATTTAGATATTA AACGATATTTAAATATACATAAAGATATATATTTGGGTGAGCGATTCCTTAAAC GAAATTGAGATTAAGGAGTCGCTCTTTTTTATGTATAAAAACAATCATGCAAAT CATTCAAATCATTTGGAAAATCACGATTTAGACAATTTTTCTAAAACCGGCTAC TCTAATAGCCGGTTGTAA
NP Fusion 4	AACAAAATAAAAAGGAGTCGCTCACGAACCTAACCTGGCATGTGAACGACATC ATTCAAAGAAAAAAACACTGAGTTGTTTTTATAATCTTGTATATTTAGATATTAA ACGATATTTAAATATACATAAAGATATATATTTGGGTGAGCGATTCCTTAAACG AAATTGAGATTAAGGAGTCGCTCTTTTTTATGTATAAAAACAATCATGCAAATC ATTCAAATCATTTGGAAAATCACGATTTAGACAATTTTTCTAAAACCGGCTACT CTAATAGCCGGTTGTAA
pT181 (SHAPE)	AACAAAATAAAAAGGAGTCGCTCACGCCCTGACCAAAGTTTGTGAACGACATC ATTCAAAGAAAAAAACACTGAGTTGTTTTTATAATCTTGTATATTTAGATATTAA ACGA
Fusion 1 (SHAPE)	AACAAAATAAAAAGGAGTCGCTCACGCTTGGCGGTGTGAACGACATCATTCAA AGAAAAAAACACTGAGTTGTTTTTATAATCTTGTATATTTAGATATTAAACGA
Fusion 2 (SHAPE)	AACAAAATAAAAAGGAGTCGCTCACGGAACTTGGCGGAACTGTGAACGACAT CATTCAAAGAAAAAAACACTGAGTTGTTTTTATAATCTTGTATATTTAGATATTA AACGA
Fusion 3 (SHAPE)	AACAAAATAAAAAGGAGTCGCTCACGCCTCGAACTTGGCGGAACGCAGTGTG AACGACATCATTCAAAGAAAAAAACACTGAGTTGTTTTTATAATCTTGTATATTT AGATATTAAACGA

Fusion 10 (SHAPE)	AACAAAATAAAAAGGAGTCGCTCACGCTTTGGCGAGTGTGAACGACATCATTC AAAGAAAAAAACACTGAGTTGTTTTTATAATCTTGTATATTTAGATATTAAACGA
Fusion 4 (SHAPE)	AACAAAATAAAAAGGAGTCGCTCACGTTCAACTTTGGCGAGTACGATGTGAAC GACATCATTCAAAGAAAAAAACACTGAGTTGTTTTTATAATCTTGTATATTTAGA TATTAAACGA
Fusion 3 L1(UU-AA) (SHAPE)	AACAAAATAAAAAGGAGTCGCTCACGCCTCGTTCTTGGCGGAACGCAGTGTG AACGACATCATTCAAAGAAAAAAACACTGAGTTGTTTTTATAATCTTGTATATTT AGATATTAAACGA
Fusion 3 L1(AA-UU) (SHAPE)	AACAAAATAAAAAGGAGTCGCTCACGCCTCGAACTTGGCGGTTCGCAGTGTG AACGACATCATTCAAAGAAAAAAACACTGAGTTGTTTTTATAATCTTGTATATTT AGATATTAAACGA
Fusion 3 L2(UC-AG) (SHAPE)	AACAAAATAAAAAGGAGTCGCTCACGCTGCGAACTTGGCGGAACGCAGTGTG AACGACATCATTCAAAGAAAAAAACACTGAGTTGTTTTTATAATCTTGTATATTT AGATATTAAACGA
Fusion 3 L2(GU-CA) (SHAPE)	AACAAAATAAAAAGGAGTCGCTCACGCCTCGAACTTGGCGGAACGAGGTGTG AACGACATCATTCAAAGAAAAAAACACTGAGTTGTTTTTATAATCTTGTATATTT AGATATTAAACGA
Fusion 4 L(UG-AC) (SHAPE)	AACAAAATAAAAAGGAGTCGCTCACGTTGTACTTTGGCGAGTACGATGTGAAC GACATCATTCAAAGAAAAAAACACTGAGTTGTTTTTATAATCTTGTATATTTAGA TATTAAACGA
Fusion 4 L(AC-UG) (SHAPE)	AACAAAATAAAAAGGAGTCGCTCACGTTCAACTTTGGCGAGTTGGATGTGAAC GACATCATTCAAAGAAAAAAACACTGAGTTGTTTTTATAATCTTGTATATTTAGA TATTAAACGA
R1 hairpin	AAAAGCAAAAACCCCGATAATCTTCTTCAACTTTGGCGAGTACGAAAAGATTA CCGGGGCCCAC
NP Fusion 1 (SHAPE)	AACAAAATAAAAAGGAGTCGCTCACGGAGGCCTTGGCGGGAGTCTGTGAACG ACATCATTCAAAGAAAAAAACACTGAGTTGTTTTTATAATCTTGTATATTTAGAT ATTAAACGA
NP Fusion 2 (SHAPE)	AACAAAATAAAAAGGAGTCGCTCACGGGGGTGTTGGCGCAGACCTGTGAACG ACATCATTCAAAGAAAAAAACACTGAGTTGTTTTTATAATCTTGTATATTTAGAT ATTAAACGA
NP Fusion 3 (SHAPE)	AACAAAATAAAAAGGAGTCGCTCACGAAGACCGCCCTCCCTGTGAACGACAT CATTCAAAGAAAAAAACACTGAGTTGTTTTTATAATCTTGTATATTTAGATATTA AACGA
NP Fusion 4 (SHAPE)	AACAAAATAAAAAGGAGTCGCTCACGAACCTAACCTGGCATGTGAACGACATC ATTCAAAGAAAAAAACACTGAGTTGTTTTTATAATCTTGTATATTTAGATATTAA ACGA
pMU720 hairpin	AAGGAAAACCCCCACTATTTTTCCTCGAACTTGGCGGAACGCAGAAAAATAAT GGGGGCCTCACAGAATAC

Supplementary Table S4: Antisense sequences

Name	Sequence
pT181	ATACAAGATTATAAAAACAACTCAGTGTTTTTTTCTTTGAATGATGTCGTTCACA AACTTTGGTCAGGGCGTGAGCGACTCCTTTTTATTT
Fusion 1	ATACAAGATTATAAAAACAACTCAGTGTTTTTTTCTTTGAATGATGTCGTTCACA CCGCCAAGCGTGAGCGACTCCTTTTTATTT
Fusion 2	ATACAAGATTATAAAAACAACTCAGTGTTTTTTTCTTTGAATGATGTCGTTCACA GTTCCGCCAAGTTCCGTGAGCGACTCCTTTTTATTT
Fusion 3	ATACAAGATTATAAAAACAACTCAGTGTTTTTTTCTTTGAATGATGTCGTTCACA CTGCGTTCCGCCAAGTTCGAGGCGTGAGCGACTCCTTTTTATTT
Fusion 10	ATACAAGATTATAAAAACAACTCAGTGTTTTTTTCTTTGAATGATGTCGTTCACA CTCGCCAAAGCGTGAGCGACTCCTTTTTATTT
Fusion 4	ATACAAGATTATAAAAACAACTCAGTGTTTTTTTCTTTGAATGATGTCGTTCACA TCGTACTCGCCAAAGTTGAACGTGAGCGACTCCTTTTTATTT
Fusion 3 L1(UU-AA)	ATACAAGATTATAAAAACAACTCAGTGTTTTTTTCTTTGAATGATGTCGTTCACA CTGCGTTCCGCCAAGAACGAGGCGTGAGCGACTCCTTTTTATTT
Fusion 3 L1(AA-UU)	ATACAAGATTATAAAAACAACTCAGTGTTTTTTTCTTTGAATGATGTCGTTCACA CTGCGAACCGCCAAGTTCGAGGCGTGAGCGACTCCTTTTTATTT
Fusion 3 L2(UC-AG)	ATACAAGATTATAAAAACAACTCAGTGTTTTTTTCTTTGAATGATGTCGTTCACA CCTCGTTCCGCCAAGTTCGAGGCGTGAGCGACTCCTTTTTATTT
Fusion 3 L2(GU-CA)	ATACAAGATTATAAAAACAACTCAGTGTTTTTTTCTTTGAATGATGTCGTTCACA CTGCGTTCCGCCAAGTTCGCAGCGTGAGCGACTCCTTTTTATTT
Fusion 4 L(UG-AC)	ATACAAGATTATAAAAACAACTCAGTGTTTTTTTCTTTGAATGATGTCGTTCACA TCGTACTCGCCAAAGTACAACGTGAGCGACTCCTTTTTATTT
Fusion 4 L(AC-UG)	ATACAAGATTATAAAAACAACTCAGTGTTTTTTTCTTTGAATGATGTCGTTCACA TCCAACTCGCCAAAGTTGAACGTGAGCGACTCCTTTTTATTT
NP Fusion 1	ATACAAGATTATAAAAACAACTCAGTGTTTTTTTCTTTGAATGATGTCGTTCACA GACTCCCGCCAAGGCCTCCGTGAGCGACTCCTTTTTATTT
NP Fusion 2	ATACAAGATTATAAAAACAACTCAGTGTTTTTTTCTTTGAATGATGTCGTTCACA GGTCTGCGCCAACACCCCCGTGAGCGACTCCTTTTTATTT
NP Fusion 3	ATACAAGATTATAAAAACAACTCAGTGTTTTTTTCTTTGAATGATGTCGTTCACA GGGAGGGCGGTCTTCGTGAGCGACTCCTTTTTATTT
NP Fusion 4	ATACAAGATTATAAAAACAACTCAGTGTTTTTTTCTTTGAATGATGTCGTTCACA TGCCAGGTTAGGTTCGTGAGCGACTCCTTTTTATTT

Supplementary Table S5: Oligonucleotides used for in-cell SHAPE-Seq. Abbreviations within primer sequences are as follows: '/5Biosg/' is a 5' biotin moiety, '/5Phos/' is a 5' monophosphate group, '/3SpC3/' is a 3' 3-carbon spacer group, VIC and NED are fluorophores (ABI), and asterisks indicate a phosphorothioate backbone modification.

**Reverse Transcription**	
ECK120051404 Terminator (ECK404)	/5Biosg/TTTATCGGCCGAAGCAGGTAG
**Adapter Ligation**	
A_adapter_b (A_b) (ssDNA adapter)	/5Phos/AGATCGGAAGAGCACACGTCTGAACTCCAGTCAC/3SpC3/
**Fluorescent Quality Analysis**	
Reverse QA primer (+)	VIC-GTGACTGGAGTTCAGACGTGTGCTC
Reverse QA primer (-)	NED-GTGACTGGAGTTCAGACGTGTGCTC
**Primers for Building dsDNA Libraries**	
ECK404 (+) selection primer (forward)	CTTTCCCTACACGACGCTCTTCCGATCTRRRYtTTATCGGCCGAAGCAGGTAgA*G*G*C
ECK404 (-) selection primer (forward)	CTTTCCCTACACGACGCTCTTCCGATCTYYYRtTTATCGGCCGAAGCAGGTAgA*G*G*C
PE_forward†	AATGATACGGCGACCACCGAGATCTACACTCTTTCCCTACACGACGCTCTTCCGATCT
**Illumina Multiplexing Primers** (Oligonucleotide sequences © 2007-2013 Illumina, Inc. All rights reserved.)	
Illumina Index #1†	CAAGCAGAAGACGGCATACGAGATCGTGATGTGACTGGAGTTCAGACGTGTGCTC
Illumina Index #2†	CAAGCAGAAGACGGCATACGAGATACATCGGTGACTGGAGTTCAGACGTGTGCTC
Illumina Index #3†	CAAGCAGAAGACGGCATACGAGATGCCTAAGTGACTGGAGTTCAGACGTGTGCTC
Illumina Index #4†	CAAGCAGAAGACGGCATACGAGATTGGTCAGTGACTGGAGTTCAGACGTGTGCTC
Illumina Index #5†	CAAGCAGAAGACGGCATACGAGATCACTGTGTGACTGGAGTTCAGACGTGTGCTC
Illumina Index #6†	CAAGCAGAAGACGGCATACGAGATATTGGCGTGACTGGAGTTCAGACGTGTGCTC
Illumina Index #7†	CAAGCAGAAGACGGCATACGAGATGATCTGGTGACTGGAGTTCAGACGTGTGCTC
Illumina Index #8†	CAAGCAGAAGACGGCATACGAGATTCAAGTGTGACTGGAGTTCAGACGTGTGCTC
Illumina Index #9†	CAAGCAGAAGACGGCATACGAGATCTGATCGTGACTGGAGTTCAGACGTGTGCTC
Illumina Index #10†	CAAGCAGAAGACGGCATACGAGATAAGCTAGTGACTGGAGTTCAGACGTGTGCTC
Illumina Index #11†	CAAGCAGAAGACGGCATACGAGATGTAGCCGTGACTGGAGTTCAGACGTGTGCTC
Illumina Index #12†	CAAGCAGAAGACGGCATACGAGATTACAAGGTGACTGGAGTTCAGACGTGTGCTC
Illumina Index #13†	CAAGCAGAAGACGGCATACGAGATTTGACTGTGACTGGAGTTCAGACGTGTGCTC
Illumina Index #14†	CAAGCAGAAGACGGCATACGAGATGGAACTGTGACTGGAGTTCAGACGTGTGCTC
Illumina Index #15†	CAAGCAGAAGACGGCATACGAGATTGACATGTGACTGGAGTTCAGACGTGTGCTC
Illumina Index #16†	CAAGCAGAAGACGGCATACGAGATGGACGGGTGACTGGAGTTCAGACGTGTGCTC
Illumina Index #18†	CAAGCAGAAGACGGCATACGAGATGCGGACGTGACTGGAGTTCAGACGTGTGCTC

†Oligonucleotide sequences © 2007-2013 Illumina, Inc. All rights reserved.

Supplementary Table S6. RMDB data deposition table. SHAPE-Seq reactivity spectra generated in this work is freely available from the RNA Mapping Database (RMDB) (http://rmdb.stanford.edu/repository/), accessible using the RMDB ID numbers listed in the table below.

Name	RMDB ID	Contents	Figure(s) used in
Fusion 1	FUS01_1M7_0001	Triplicate data of Fusion 1 sensing hairpin	2, S2
Fusion 2	FUS02_1M7_0001	Triplicate data of Fusion 2 sensing hairpin	2, S2, S5
Fusion 3	FUS03_1M7_0001	Triplicate data of Fusion 3 sensing hairpin	2, 3, 4, 5, S2, S6, S8
Fusion 4	FUS04_1M7_0001	Triplicate data of Fusion 4 sensing hairpin	2, 3, 5, S4
Fusion 10	FUS10_1M7_0001	Triplicate data of Fusion 10 sensing hairpin	2, S4, S5
Fusion 3 L1(UU-AA)	FUS3L1A_1M7_0001	Triplicate data of Fusion 3 L1(UU-AA) sensing hairpin	3
Fusion 3 L1(AA-UU)	FUS3L1B_1M7_0001	Single data of Fusion 3 L1(AA-UU) sensing hairpin	S6
Fusion 3 L2(UC-AG)	FUS3L2B_1M7_0001	Single data of Fusion 3 L2(UC-AG)	S6
Fusion 3 L2(GU-CA)	FUS3L2A_1M7_0001	Triplicate data of Fusion 3 L2(GU-CA) sensing hairpin	3, 4, S5, S8
Fusion 4 L(UG-AC)	FUS4LA_1M7_0001	Triplicate data of Fusion 4 L(UG-AC) sensing hairpin	3, S5
Fusion 4 L(AC-UG)	FUS4LB_1M7_0001	Single data of Fusion 4 L(AC-UG) sensing hairpin	S7
NP Fusion 1	NPFUS1_1M7_0001	Triplicate data of NP Fusion 1 sensing hairpin	6, S13
NP Fusion 2	NPFUS2_1M7_0001	Triplicate data of NP Fusion 2 sensing hairpin	6, S13
NP Fusion 3	NPFUS3_1M7_0001	Triplicate data of NP Fusion 3 sensing hairpin	S12
NP Fusion 4	NPFUS4_1M7_0001	Triplicate data of NP Fusion 4 sensing hairpin	S12
pMU720 hairpin	PMU720_1M7_0001	Triplicate data of pMU720 regulator sensing hairpin	5, S11
R1 hairpin	R1HP1_1M7_0001	Quadruple data of R1 regulator sensing hairpin	5, S11

Supplementary Table S7. A thermodynamic folding analysis of the ON and OFF states predicts that all fusions should be functional. Free energies of the ON, OFF (no antisense), and OFF (with antisense) were calculated using RNAstructure (Reuter and Mathews 2010) and are reported in kcal/mol. The ON structure was obtained by forced pairing of the antiterminator with the 5' half of the terminator stem using the RNAStructure `fold` utility with default parameters. Similarly, the OFF (no antisense) structure is the lowest free energy structure where the complete terminator and poly U are formed. The OFF-with-antisense free energy was calculated using the `duplex` utility by also including the complete antisense sequence (without terminator). All of these analysis predict that OFF (with antisense) is much more stable than OFF (no antisense), indicating that from a thermodynamic perspective all fusions should be functional.

Attenuator	ON	OFF (no antisense)	OFF (with antisense)
Fusion 1	-50.1	-54.6	-173.6
Fusion 2	-48.2	-52.7	-184.4
Fusion 3	-54.6	-55.5	-204.8
Fusion 3 L1(UU-AA)	-58	-61.4	-204.8
Fusion 3 L2(GU-CA)	-56	-63.1	-204.8

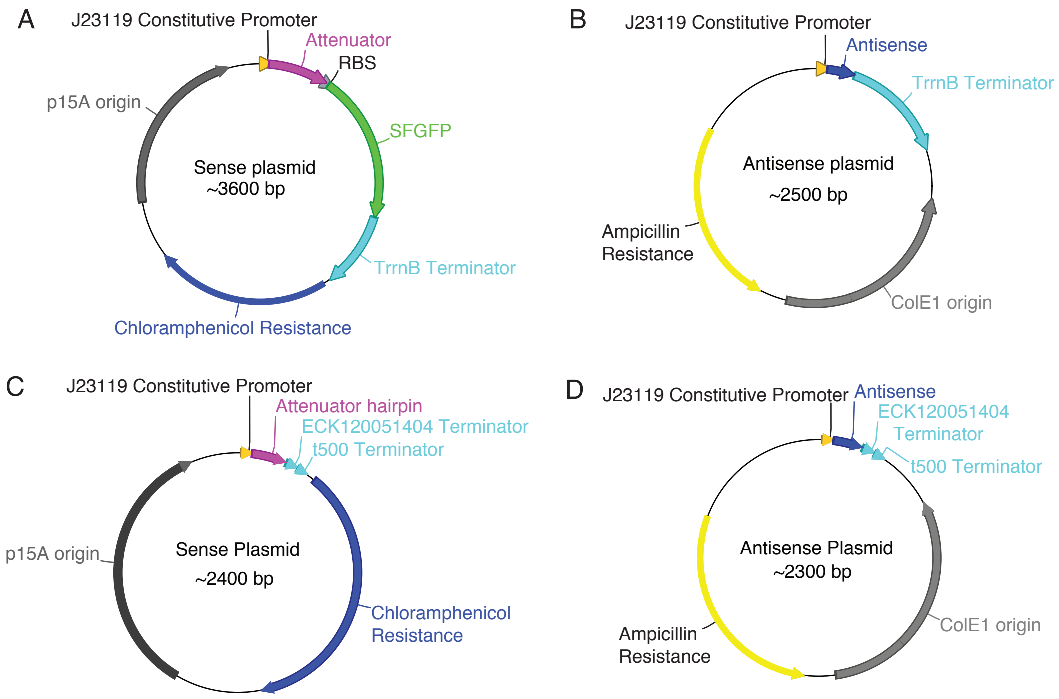

Supplementary Figure S1. Plasmid architecture for (A) Attenuator-SFGFP constructs for functional testing. (B) Antisense constructs for functional testing. (C) Truncated attenuator constructs for in-cell SHAPE-Seq. (D) Antisense constructs for functional testing or in-cell SHAPE-Seq. Specific sequences can be found in Supplementary Tables S2-S4. The no-antisense control plasmid lacked an antisense coding sequence.

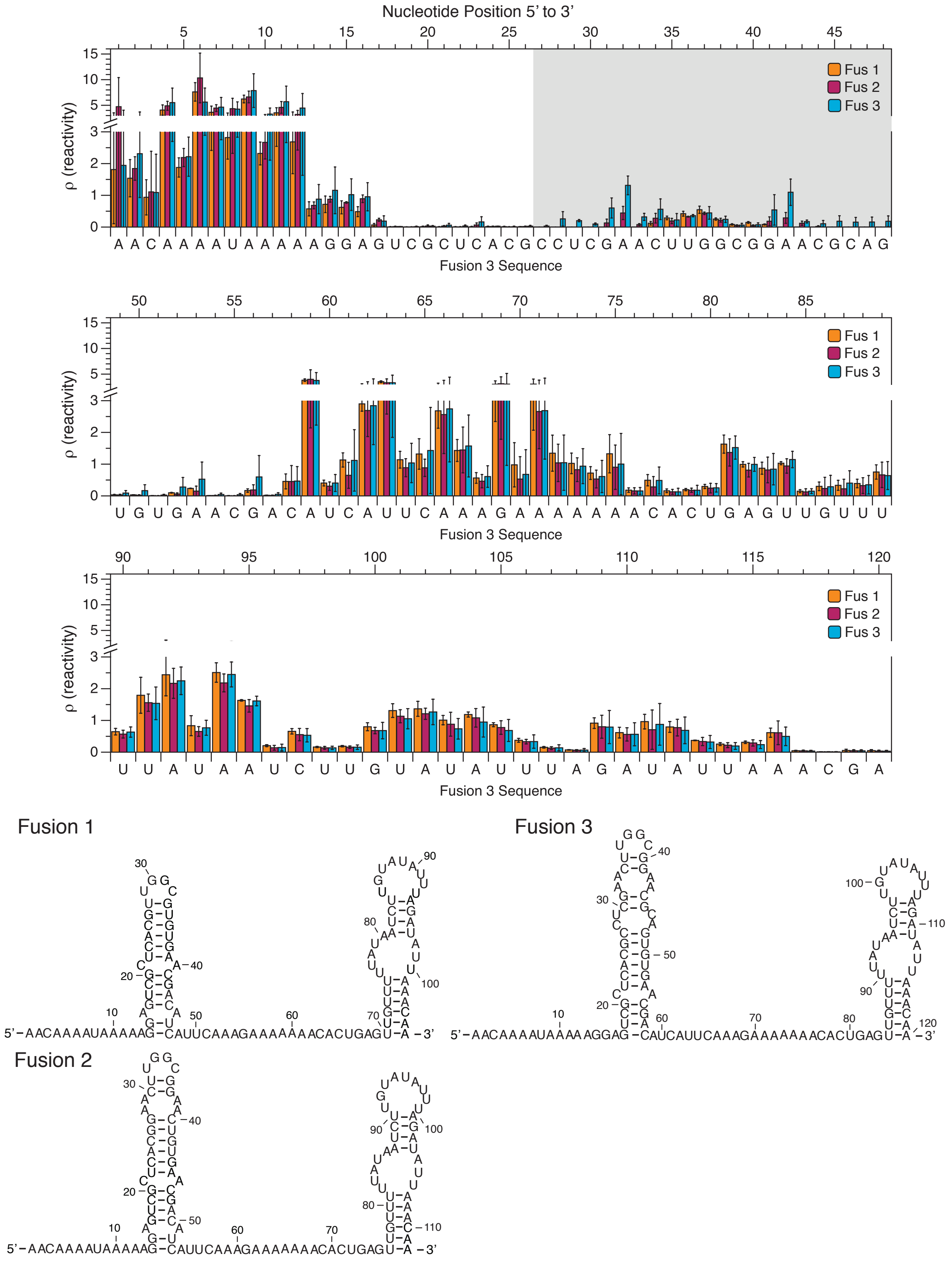

Nucleotide Position 5' to 3'
ρ (reactivity)
Fus 1
Fus 2
Fus 3
Fusion 3 Sequence
Fusion 1
Fusion 2
Fusion 3
5'-AACAAAAUAAAAAG-CAUUCAAAGAAAAAAACACUGAGU-A-3'
5'-AACAAAAUAAAAAG-CAUUCAAAGAAAAAAACACUGAGU-A-3'
5'-AACAAAAUAAAAAGGAG-CAUCAUUCAAAGAAAAAAACACUGAGU-A-3'

Supplementary Figure S2. Full comparison of in-cell SHAPE-Seq reactivity spectra for Fusions 1-3. The common sequence from the pT181 attenuator is nucleotides 1-26 and 49-120. Shaded region indicates the fusion region shown in Figure 2. The Fusion 3 sequence is used for the comparison. Nucleotide positions that are not included in Fusions 1 or 2 are left without data. Reactivity spectra represent an average of three independent in-cell SHAPE-Seq experiments with error bars representing standard deviations at each nucleotide. Secondary structures are in-cell SHAPE-constrained predictions (see Materials and Methods).

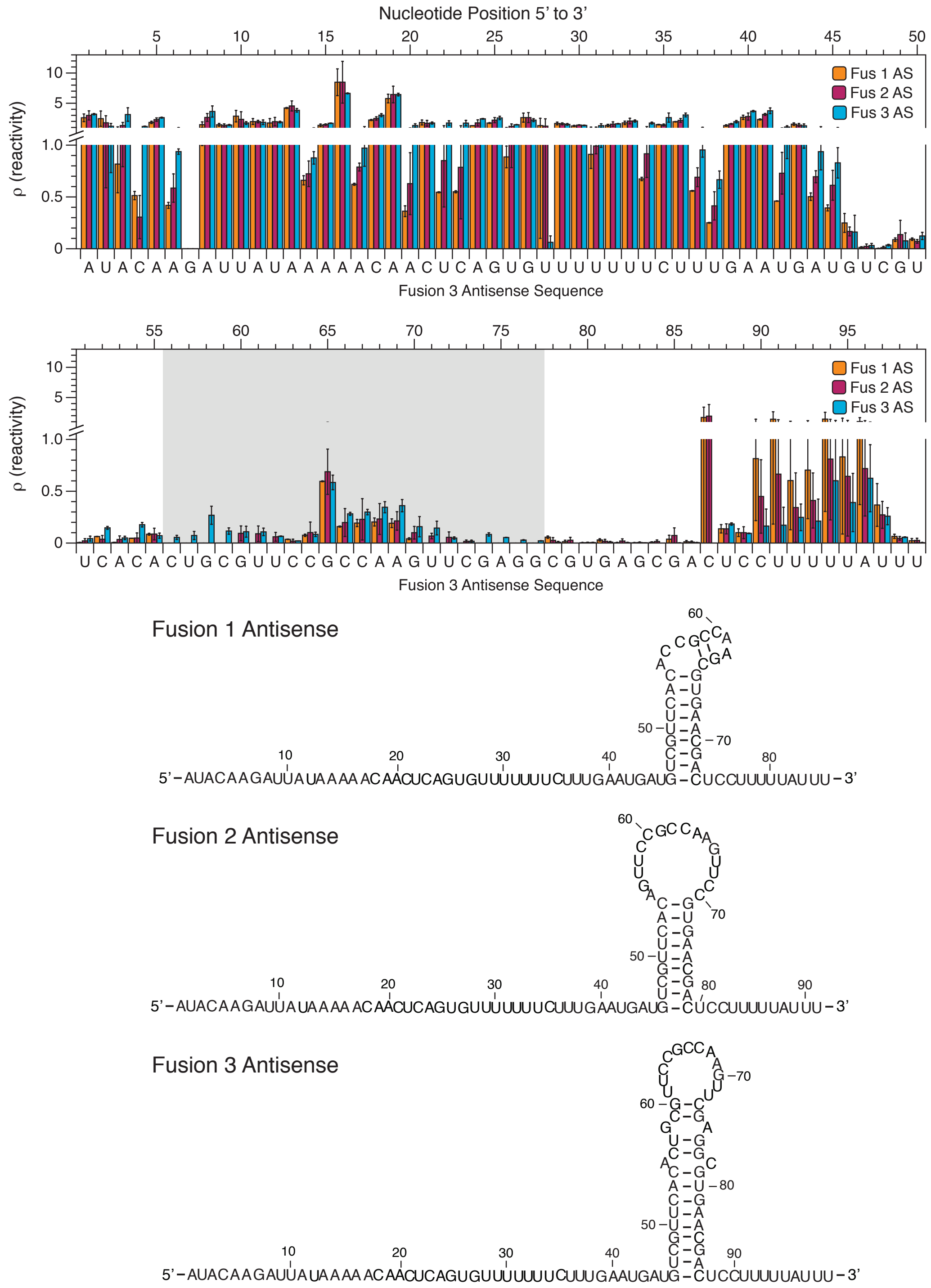

Supplementary Figure S3. In-cell SHAPE-Seq reactivity comparison for Fusions 1-3 antisense RNAs (AS). The common sequence from the pT181 antisense is nucleotides 1-55 and 78-99. The shaded region indicates the pMU720 sequence included for each Fusion. The Fusion 3 antisense sequence is used for the comparison. Nucleotide positions that are not included in Fusions 1 or 2 are left without data. Reactivity spectra represent an average of two independent in-cell SHAPE-Seq experiments with error bars representing the high and low value for each nucleotide. Secondary structures are in-cell SHAPE-constrained predictions (see Materials and Methods).

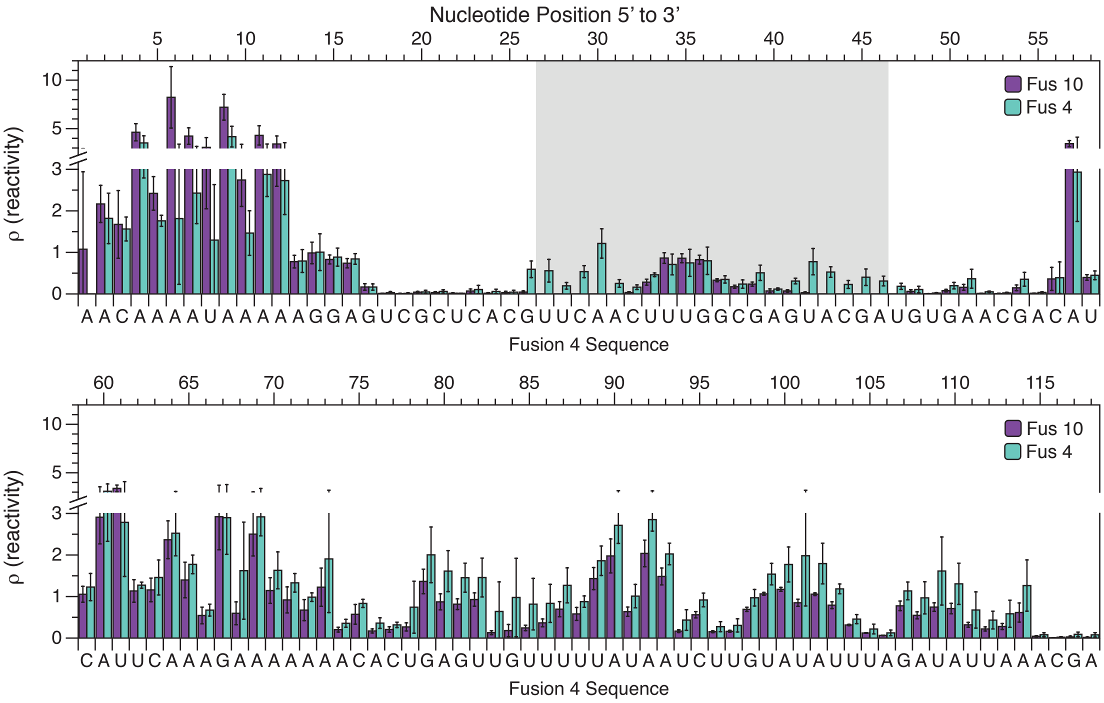

Supplementary Figure S4. Full comparison of in-cell SHAPE-Seq reactivity spectra for Fusions 10 and 4. The common sequence from the pT181 attenuator is nucleotides 1-26 and 47-118. Shaded region indicates the fusion region shown in Figure 2. The Fusion 4 sequence is used for the comparison. Nucleotide positions that are not included in Fusion 10 are left without data. Reactivity spectra represent an average of three independent in-cell SHAPE-Seq experiments with error bars representing standard deviations at each nucleotide.

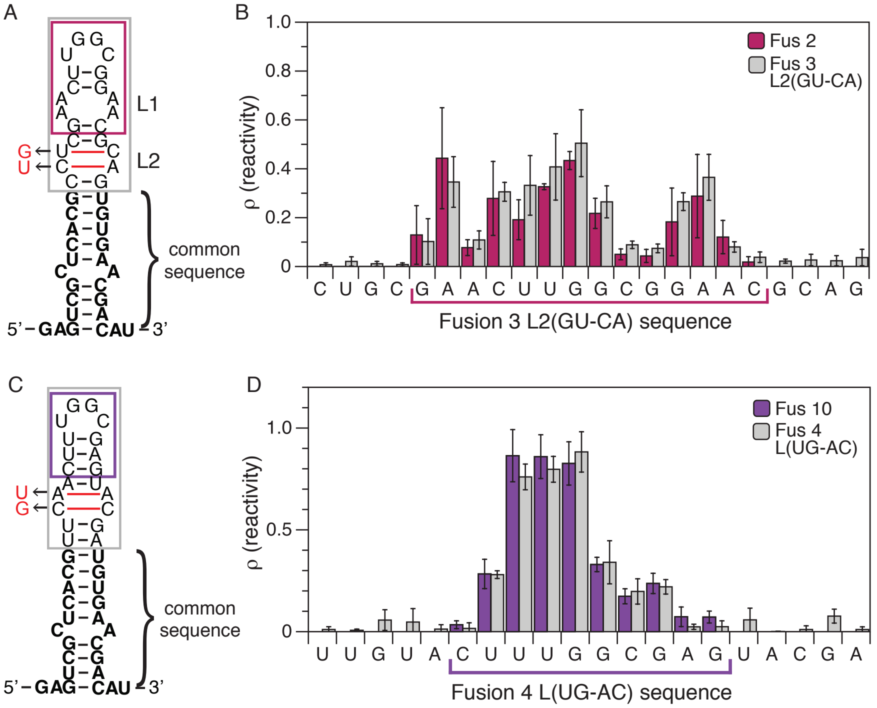

Supplementary Figure S5. Comparison of interior loop closures to non-functional fusions. (A) In-cell SHAPE-constrained secondary structure prediction of the Fusion 3 hairpin indicating mutations to close the lower interior loop L2(GU-CA). Boxed region indicates nucleotides shown in the reactivity spectra in (B). The nucleotides included in Fusion 2 are in the colored box. (B) In-cell SHAPE-Seq reactivity spectra comparing Fusion 2 and Fusion 3 L2(GU-CA). The data is the same as represented in Figures 2C and 3F respectively. (C) In-cell SHAPE-constrained secondary structure prediction of the Fusion 4 hairpin indicating mutations to close the interior loop L(UG-AC). Boxed region indicates nucleotides shown in the reactivity spectra in D. The nucleotides included in Fusion 10 are in the colored box. (D) In-cell reactivity spectra comparing Fusion 10 and Fusion 4 L(UG-AC). The data is the same as represented in Figures 2F and 3I respectively.

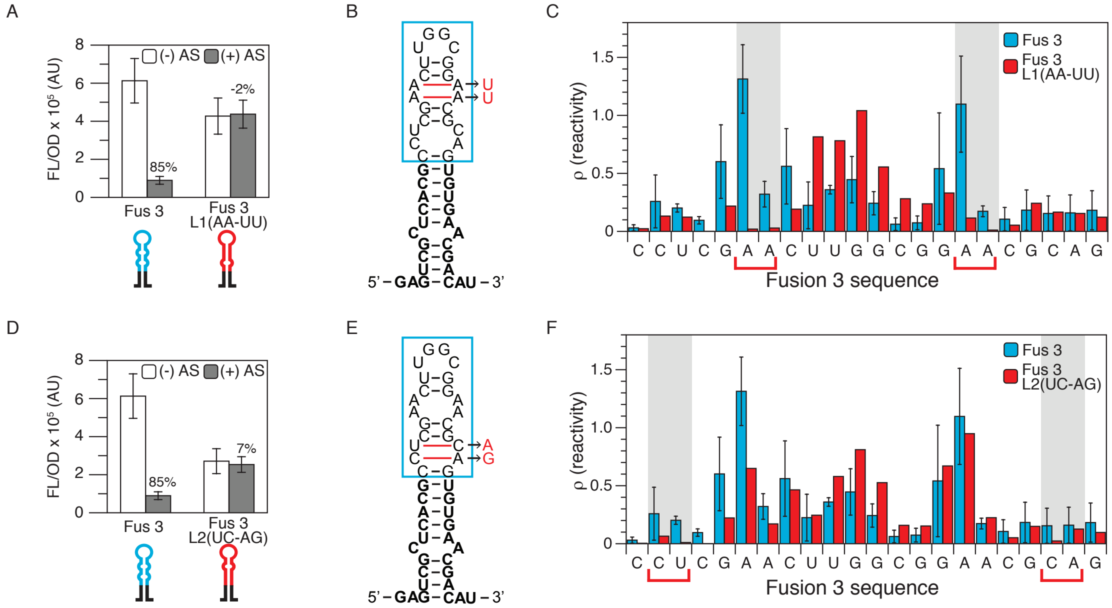

Supplementary Figure S6. Mutations to the 3' side of Fusion 3 interior loops. Data complementary to Figure 3. The Fusion 3 data is the same as represented in Figure 3. (A) Functional characterization of Fusion 3 and the Fusion 3 L1 mutant that closes the top interior loop. Average fluorescence (FL/OD) of *E. coli* TG1 cells with (+ AS) or without (- AS) antisense RNA. Error bars represent standard deviations of nine biological replicates. (B) In-cell SHAPE--constrained secondary structure prediction of the Fusion 3 hairpin indicating mutations to close the upper interior loop (L1, AA-UU). Boxed region indicates nucleotides shown in the reactivity spectra in (C). (C) In-cell SHAPE-Seq reactivity comparison for Fusion 3 and Fusion 3 L1(AA-UU). A single in-cell SHAPE-Seq experiment was performed for Fusion 3 L1(AA-UU). Shaded regions and colored brackets indicate nucleotides of the mutated interior loop. (D, E, F) As in (A, B, C) but for the Fusion 3 L2 mutant that closes the bottom interior loop.

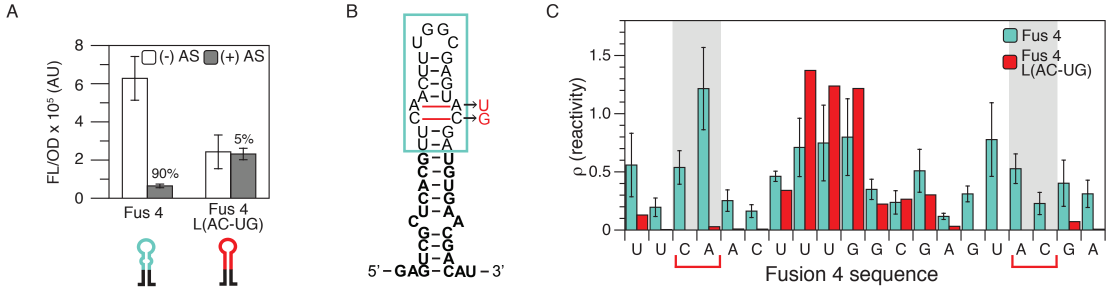

Supplementary Figure S7. Mutations to the 3' side of the Fusion 4 interior loop. Data complementary to Figure 3. The Fusion 4 data is the same as represented in Figure 3. (A) Functional characterization of Fusion 4 and the Fusion 4 mutant that closes the interior loop. Average fluorescence (FL/OD) of *E. coli* TG1 cells with (+ AS) or without (- AS) antisense RNA. Error bars represent standard deviations of nine biological replicates. (B) In-cell SHAPE-constrained secondary structure prediction of the Fusion 4 hairpin indicating mutations to close the interior loop L(AC-UG). Boxed region indicates nucleotides shown in the reactivity spectra in (C). (C) In-cell SHAPE-Seq reactivity comparison for Fusion 4 and Fusion 4 L(AC-UG). A single in-cell SHAPE-Seq experiment was performed for Fusion 4 L(AC-UG). Shaded regions and colored brackets indicate nucleotides of the mutated interior loop.

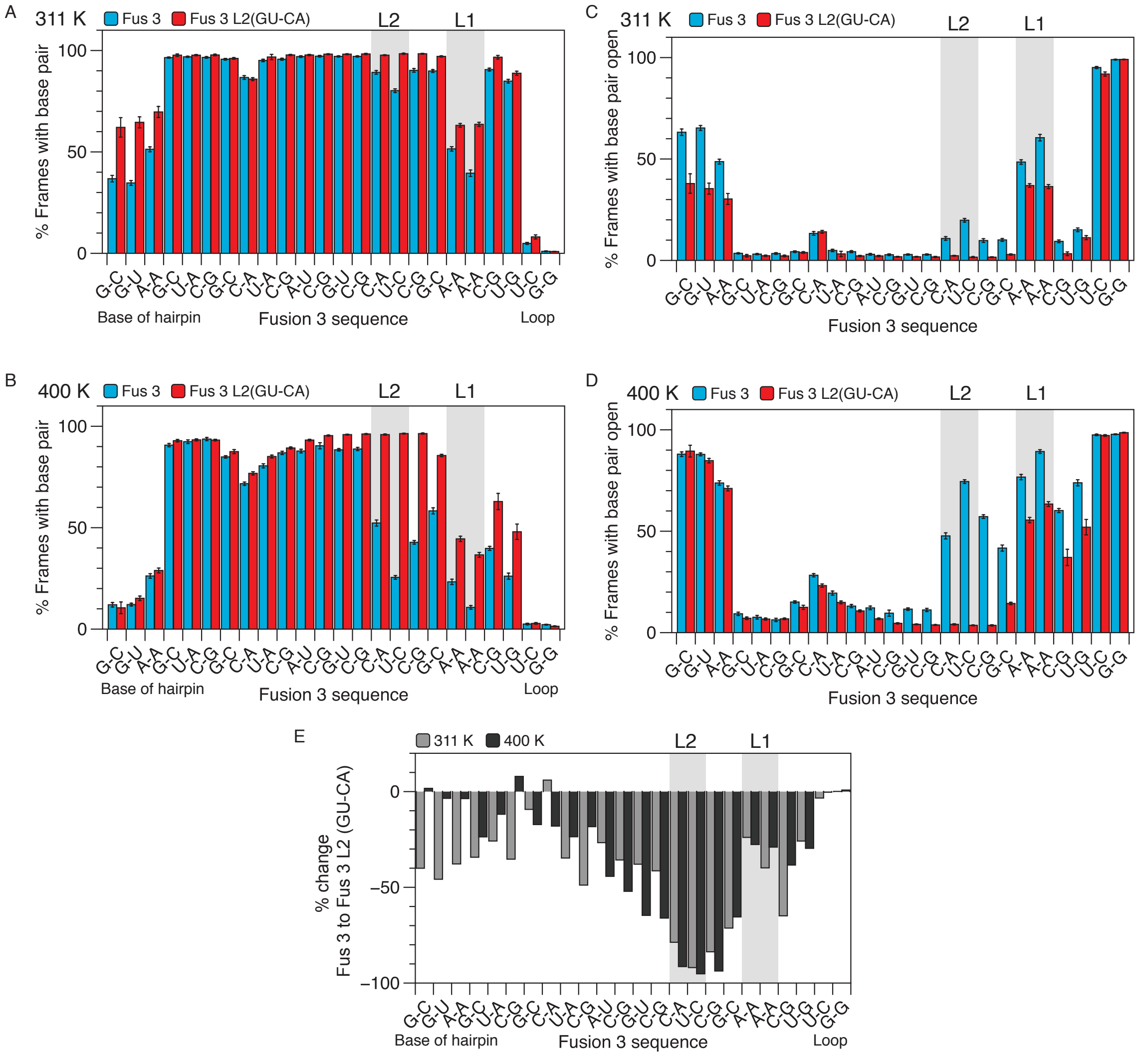

Supplementary Figure S8. Percent base pair occupancies from molecular dynamics simulations performed at (A) 311 K and (B) 400 K show the increased stability in the fusion region of the interior loop mutant Fusion 3 L2(GU-CA) compared to Fusion 3. Simulation data converted to percent frames with bases unpaired, or open, for (C) 311K and (D) 400K to allow for comparison to SHAPE-Seq reactivities. (E) Comparison of percent change in base pair occupancy at 311 K and 400 K. Percent base pair occupancies were first converted into percentages of frames in which each base pair was not occupied (C and D). This was then used to calculate a percent change in this value from Fusion 3 to Fusion 3 L2(GU-CA) at each base pair. Shaded regions indicate the L1 and L2 interior loops.

A

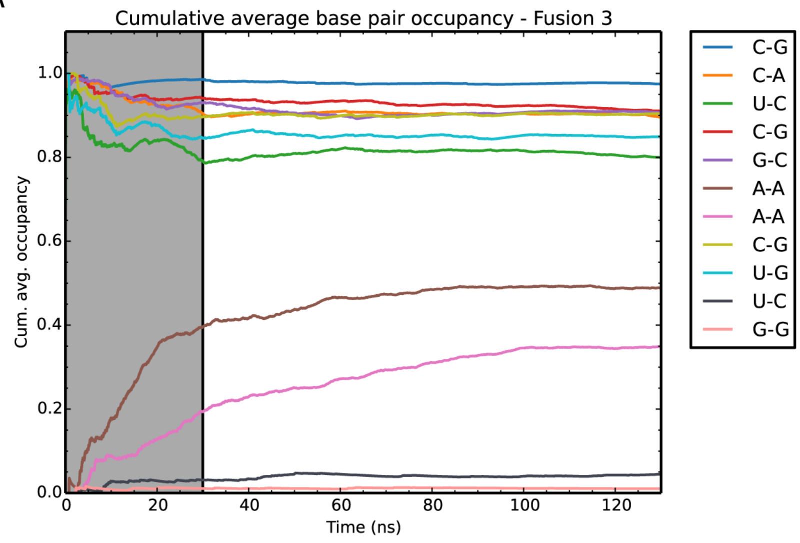

B

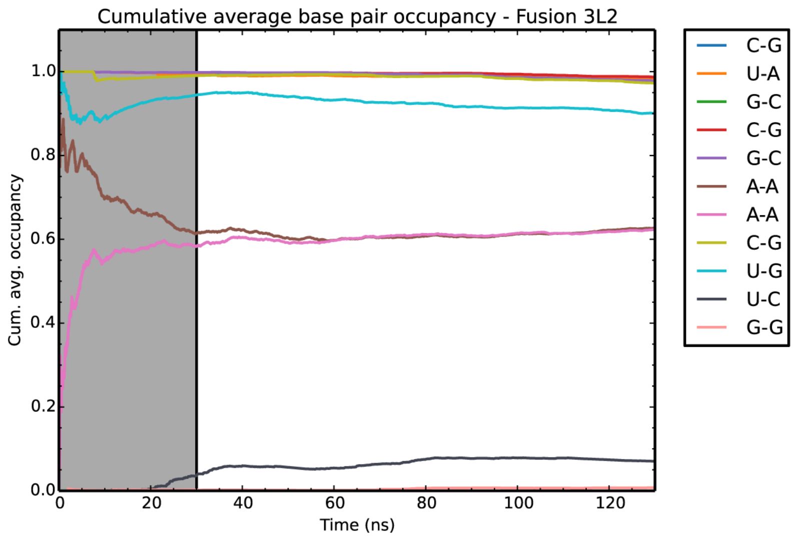

Supplementary Figure S9. The simulation cumulative average base pair occupancy is shown for A) fusion 3 and B) fusion 3L2 at 311K. The first 30 ns (the grayed region) was discarded as equilibration while the following 100 ns was considered converged and used in the calculation of the reported base pair occupancies.

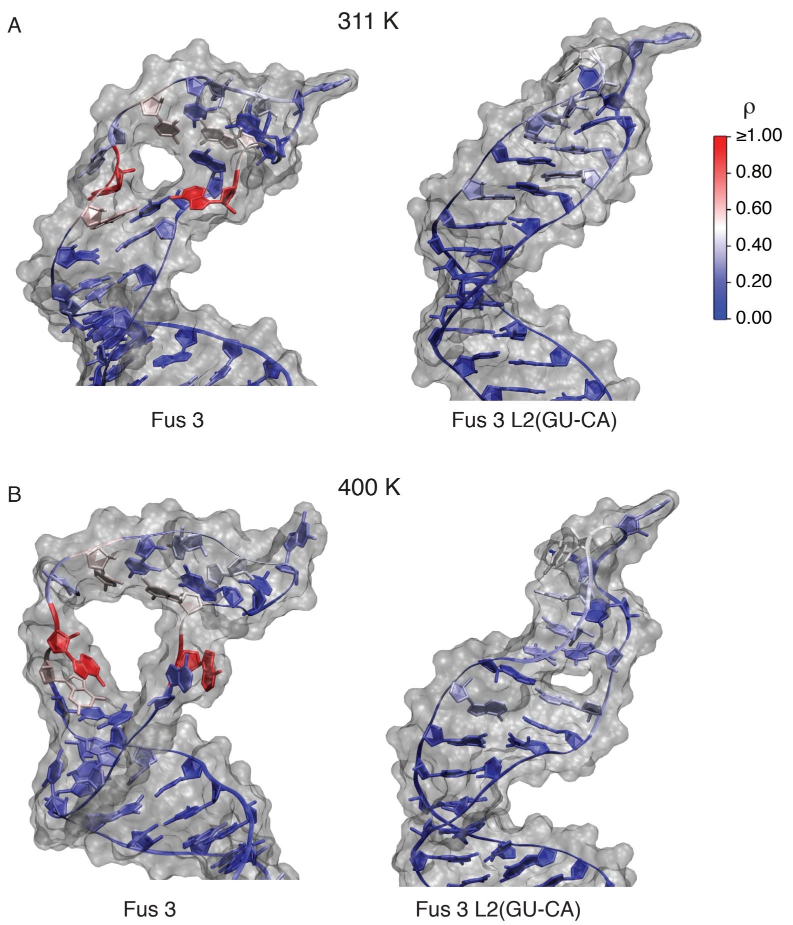

Supplementary Figure S10. Representative simulation structures are shown at (A) 311 K and (B) 400 K for Fusion 3 and Fusion 3 L2(GU-CA). Ribbon representations of nucleic acid backbone and bases are colored according to in-cell SHAPE-Seq reactivities from Figure 3F. Solvent accessible surface representations, shown in transparent gray, depict the points contacted by a spherical probe of 1.4Å radius rolled across the van der Waals radii of the RNA atoms. Images generated using VMD software (Humphrey et al. 1996).

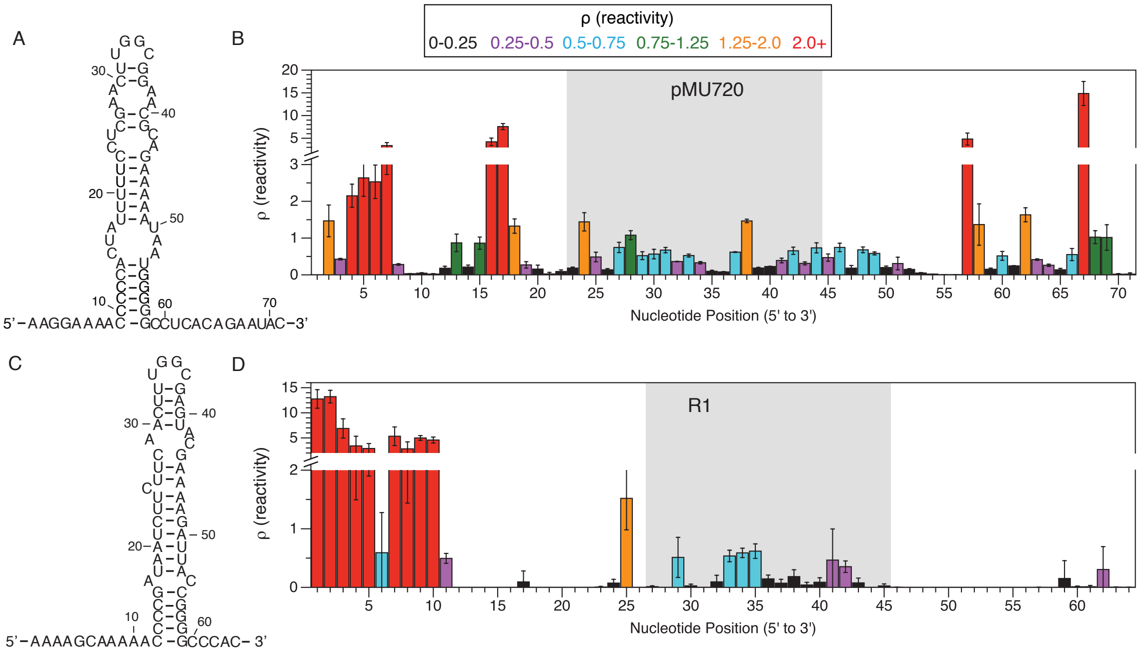

Supplementary Figure S11. Full in-cell SHAPE-Seq reactivity profiles for natural translational regulator hairpins. (A) In-cell SHAPE-constrained secondary structure prediction of the pMU720 hairpin with in-cell SHAPE-Seq reactivity profile in (B). (C) In-cell SHAPE-constrained secondary structure prediction of the R1 hairpin with in-cell SHAPE-Seq reactivity profile in (D). Reactivity spectra represent an average of three independent in-cell SHAPE-Seq experiments with error bars representing standard deviations at each nucleotide. Shaded regions indicate the data presented in Figure 5.

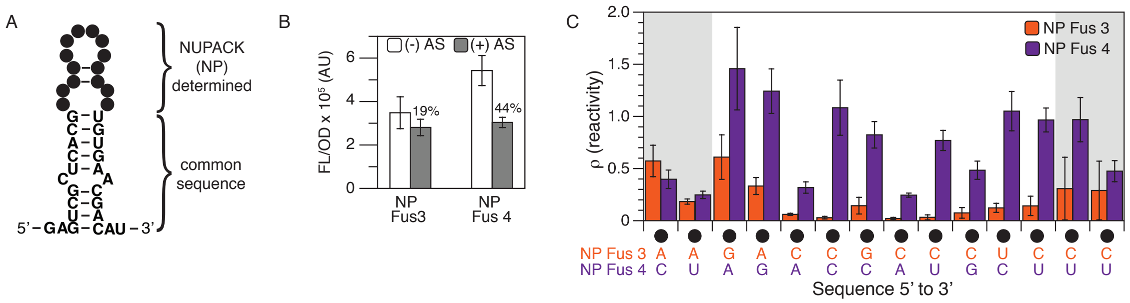

Supplementary Figure S12. Using NUPACK to design additional chimeric attenuators with defined interior loops. (A) NUPACK (Zadeh et al. 2011) design constraints. The nucleotides specified in the base of the hairpin are the same as those in the fusions from this study. Filled circles represent nucleotides that NUPACK was allowed to design. (B) Functional characterization of two NUPACK (NP) designed fusions. Average fluorescence (FL/OD) of *E. coli* TG1 cells with (+ AS) or without (- AS) antisense RNA. Error bars represent standard deviations of nine biological replicates. (C) In-cell SHAPE-Seq reactivity spectra for the upper portion of NP Fusion 3 and 4 hairpin stems. Shaded regions indicate nucleotides in the interior loop. Reactivity spectra represent an average of three independent in-cell SHAPE-Seq experiments with error bars representing standard deviations at each nucleotide.

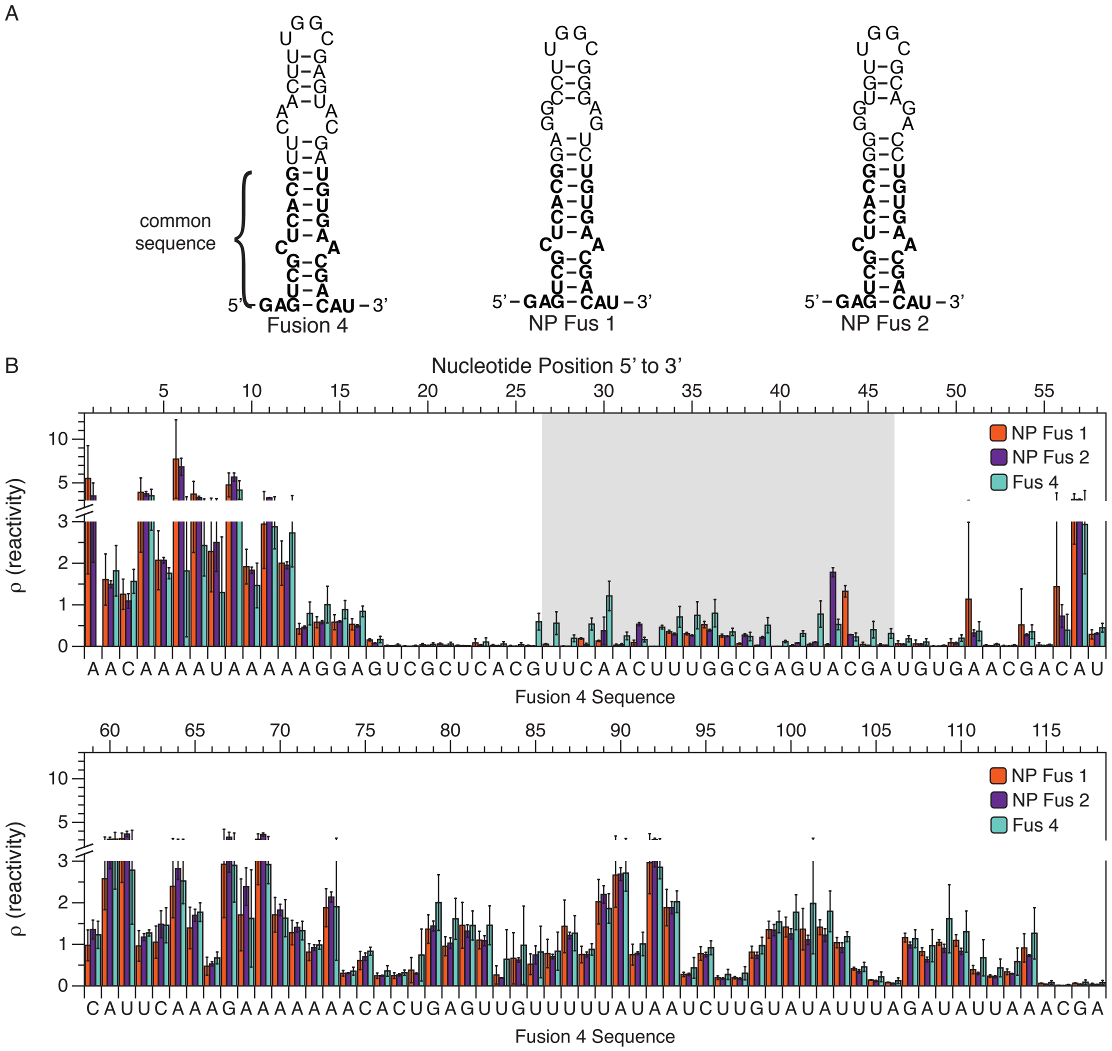

Supplementary Figure S13. (A) Secondary structure prediction of the Fusion 4, NUPACK Fusion 1 and 2 hairpins. (B) Full in-cell SHAPE-Seq reactivity profiles for NUPACK fusions compared to Fusion 4. The common sequence from the pT181 attenuator is nucleotides 1-26 and 47-118. Shaded region indicates the fusion region shown in Figure 2 and 6. The Fusion 4 sequence is used for the comparison. Nucleotide positions that are not included in NP Fusion 1 and 2 are left without data. Reactivity spectra represent an average of three independent in-cell SHAPE-Seq experiments with error bars representing standard deviations at each nucleotide.

Supplementary Note. Code used to design *in silico* attenuators using the NUPACK webservers (Zadeh et al. 2011).

```
# NUPACK web server design algorithm
material = rna
temperature[C] = 37
trials = 2
sodium[M] = 1.0
dangles = some
allowmismatch = true

# target structure
structure stickfigure = .............((.((((.(((((((..(((....)))..))))))).)))).)).......

#sequence domains
domain a = AACAAAATAAAAAGGAGTCGCTCACG
domain b = N6
domain c = TTGGCG
domain d = N6
domain e = TGTGAACGACATCATTCAAA

stickfigure.seq = a b c d e

stickfigure.stop = 10.0
```

Supplementary Movie 1. Movie of a 2 ns segment from REMD simulations of Fusion 3. These trajectories were generated by following the dynamics of an initial conformation, including exchanges across neighboring temperature replicas. This process results in a physically contiguous trajectory in which the simulation temperature is free to gradually vary in the REMD range (290.00 K to 435.10 K). Ribbon representations of nucleic backbone and bases are colored according to in-cell SHAPE-Seq reactivities from Figure 3F. The surface, shown in transparent gray, follows the contour of a constant global atomic density generated using the VMD software (Chen and García 2013; Humphrey et al. 1996) qsurf representation.

Supplementary Movie 2. Movie of a 2 ns segment from REMD simulations of Fusion 3 L2(GU-CA). As in Supplementary Movie 1.

References

Aaqvist J. 1990. Ion-water interaction potentials derived from free energy perturbation simulations. *THe Journal of Physical chemistry* **94**: 8021–8024.

Cheatham TEI, Miller JL, Fox T, Darden TA, Kollman PA. 1995. Molecular Dynamics Simulations on Solvated Biomolecular Systems: The Particle Mesh Ewald Method Leads to Stable Trajectories of DNA, RNA, and Proteins. *J Am Chem Soc* **117**: 4193–4194.

Chen AA, García AE. 2013. High-resolution reversible folding of hyperstable RNA tetraloops using molecular dynamics simulations. *Proc Natl Acad Sci USA* **110**: 16820–16825.

Chen AA, Pappu RV. 2007. Parameters of Monovalent Ions in the AMBER-99 Forcefield: Assessment of Inaccuracies and Proposed Improvements. *J Phys Chem B* **111**: 118884–118887.

Chen Y-J, Liu P, Nielsen AAK, Brophy JAN, Clancy K, Peterson T, Voigt CA. 2013. Characterization of 582 natural and synthetic terminators and quantification of their design constraints. *Nat Methods* **10**: 659–664.

García AE, Herce H, Paschek D. 2006. Simulations of temperature and pressure unfolding of peptides and proteins with replica exchange molecular dynamics. Vol. 2 of, pp. 83–95, Annu Rep Comput Chem.

Humphrey W, Dalke A, Schulten K. 1996. VMD: Visual molecular dynamics. *Journal of molecular graphics* **14**: 33–38.

Jorgensen WL, Chandrasekhar J, Madura JD, Impey RW, Klein ML. 1983. Comparison of simple potential functions for simulating liquid water. *J Chem Phys* **79**: 926.

Nishikawa S, Huang H, Jordan F. 2000. Structural Effect of Nucleotides on Syn−Anti Glycosyl Isomerization Kinetics by Ultrasonic Relaxation Methods. *J Phys Chem B* **104**: 1391–1394.

Parisien M, Major F. 2008. The MC-Fold and MC-Sym pipeline infers RNA structure from sequence data. *Nature* **452**: 51–55.

Patriksson A, van der Spoel D. 2008. A temperature predictor for parallel tempering simulations. *Physical Chemistry Chemical Physics* **10**: 2073–2077.

Pronk S, Pall S, Schulz R, Larsson P, Bjelkmar P, Apostolov R, Shirts MR, Smith JC, Kasson PM, van der Spoel D, et al. 2013. GROMACS 4.5: a high-throughput and highly parallel open source molecular simulation toolkit. *Bioinformatics* **29**: 845–854.

Reuter JS, Mathews DH. 2010. RNAstructure: software for RNA secondary structure prediction and analysis. *BMC Bioinformatics* **11**: 129.

Rinnenthal J, Klinkert B, Narberhaus F, Schwalbe H. 2010. Direct observation of the temperature-induced melting process of the Salmonella fourU RNA thermometer at base-pair resolution. *Nucleic acids research* **38**: 3834–3847.

Sorin EJ, Pande VS. 2005. Exploring the Helix-Coil Transition via All-Atom Equilibrium Ensemble Simulations. *Biophys J* **88**: 2472–2493.

Wang J, Cieplak P, Kollman PA. 2000. How well does a restrained electrostatic potential (RESP) model perform in calculating conformational energies of organic and biological molecules? *J Comput Chem* **21**: 1049–1074.

Zadeh JN, Wolfe BR, Pierce NA. 2011. Nucleic acid sequence design via efficient ensemble defect optimization. *J Comput Chem* **32**: 439–452.
